# Neutrophil Extracellular Traps Contain Calprotectin, a Cytosolic Protein Complex Involved in Host Defense against *Candida albicans*

**DOI:** 10.1371/journal.ppat.1000639

**Published:** 2009-10-30

**Authors:** Constantin F. Urban, David Ermert, Monika Schmid, Ulrike Abu-Abed, Christian Goosmann, Wolfgang Nacken, Volker Brinkmann, Peter R. Jungblut, Arturo Zychlinsky

**Affiliations:** 1 Department for Cellular Microbiology, Max Planck Institute for Infection Biology, Berlin, Germany; 2 Protein Analysis Core Facility, Max Planck Institute for Infection Biology, Berlin, Germany; 3 Microscopy Core Facility, Max Planck Institute for Infection Biology, Berlin, Germany; 4 Institute for Immunology, Münster University, Münster, Germany; 5 Institute for Molecular Virology, Center for Molecular Biology of Inflammation, Münster University, Münster, Germany; UMass Medical Center, United States of America

## Abstract

Neutrophils are the first line of defense at the site of an infection. They encounter and kill microbes intracellularly upon phagocytosis or extracellularly by degranulation of antimicrobial proteins and the release of Neutrophil Extracellular Traps (NETs). NETs were shown to ensnare and kill microbes. However, their complete protein composition and the antimicrobial mechanism are not well understood. Using a proteomic approach, we identified 24 NET-associated proteins. Quantitative analysis of these proteins and high resolution electron microscopy showed that NETs consist of modified nucleosomes and a stringent selection of other proteins. In contrast to previous results, we found several NET proteins that are cytoplasmic in unstimulated neutrophils. We demonstrated that of those proteins, the antimicrobial heterodimer calprotectin is released in NETs as the major antifungal component. Absence of calprotectin in NETs resulted in complete loss of antifungal activity *in vitro*. Analysis of three different *Candida albicans in vivo* infection models indicated that NET formation is a hitherto unrecognized route of calprotectin release. By comparing wild-type and calprotectin-deficient animals we found that calprotectin is crucial for the clearance of infection. Taken together, the present investigations confirmed the antifungal activity of calprotectin *in vitro* and, moreover, demonstrated that it contributes to effective host defense against *C. albicans in vivo*. We showed for the first time that a proportion of calprotectin is bound to NETs *in vitro* and *in vivo*.

## Author Summary

Neutrophils are phagocytes that disarm and kill microbes by engulfing them. Less well characterized than their phagocytic killing mechanisms is how neutrophils cope with microbes that are too large to be internalized. Notably, neutrophils may also kill or inhibit extracellularly by releasing Neutrophil Extracellular Traps (NETs). NETs are fibers made of chromatin (histones and DNA) decorated with antimicrobial proteins. NETs ensnare and kill microbes, such as bacteria, fungi and parasites. We wanted to find out if and how NETs control pathogenic fungi that can form large filaments such as *Candida albicans*. We purified all NET-bound proteins and identified 24 of them. We found that calprotectin is the major antifungal NET-bound protein. Calprotectin was known to be antimicrobial but here we demonstrate that NET formation is a novel release mechanism for this cytoplasmic protein. The NET matrix comes in close contact with the fungi and the high local concentration of calprotectin in the NETs supports the antifungal activity. Furthermore, in mice calprotectin is essential for an efficient antifungal response to *Candida albicans* in skin, lung and systemic infections. In tissue sections from these animals we detected NETs and NET-associated calprotectin. Thus, our study gives more insights into mechanisms how the immune system copes with fungal pathogens.

## Introduction

Neutrophils are an essential component of the innate immune response since neutropenia or impairment of neutrophil function results in microbial infections that are often fatal [1]. Microbes engulfed by neutrophils are efficiently killed by reactive oxygen species (ROS) and antimicrobial proteins within vacuoles [2]. Additionally, neutrophils [3] and two other granulocytes, mast cells [4] and eosinophils [5], release web-like extracellular traps that ensnare and kill microbes.

Neutrophil Extracellular Traps (NETs) are released during a novel form of cell death that requires ROS produced by the NADPH-oxidase complex [6]. During this process, the nucleus decondenses and intracellular membranes disintegrate allowing the mixing of nuclear and cytoplasmic components. Eventually, the plasma membrane ruptures to release NETs, structures that contain chromatin and granule proteins. The overall composition of NETs has not been explored. Neutrophils of several species make NETs [7],[8],[9] and they might be important in the immune defense against bacteria and fungi [10],[11],[12],[13]. Whereas bacteria [3] and parasites [14] probably are killed by histones in NETs, in a previous study we found that purified histones did affect *Candida albicans in vitro* only poorly [13]. Thus, it remains to be determined whether histones or other antifungal effectors in NETs kill or inhibit fungi. This seems to be particularly of importance since previous reports have demonstrated that histones and histone peptides kill different fungal species such as *Cryptococcus neoformans* and *Candida tropicalis* [15],[16],[17].

Fungal pathogens, in particular *C. albicans*, cause an increasing number of severe infections with high mortality rates [18]. *C. albicans* is an opportunistic pathogen that can be part of the normal microbial flora of humans. In immunosuppressed patients the microbe can use a variety of virulence factors that enables it to exploit various host niches and to cause different diseases ranging from cutaneous to systemic infections [19]. A key characteristic of *C. albicans* is the ability to change growth morphology from budding yeast to filamentous forms: pseudohyphae and true hyphae [20]. A variety of external stimuli have been shown to induce the yeast-to-hyphae transition, such as serum, alkaline pH and temperatures above 37°C [21]. The ability to reversibly switch between different morphologies upon external stimuli appears to be essential for the virulence of *C. albicans* [22],[23].

Using a proteomic approach, we analyzed the qualitative and quantitative protein composition of NETs. We identified 24 different proteins, including the cytoplasmic calprotectin protein complex (also called Mrp8/14-complex or S100A8/A9) that has been shown previously by several groups to have potent antimicrobial properties [24],[25],[26]. S100A8 and S100A9 belong to the large group of S100 calcium-binding proteins and form a heterodimer, calprotectin, which is abundant in neutrophils, monocytes and early differentiation stages of macrophages [27]. In other cell types, such as keratinocytes and epithelial cells, the expression can be induced under inflammatory conditions [28]. The antibacterial and antifungal activity of the complex is reversible by Zn^2+^ [29] and does not require direct contact to the microbe [30],[31]. Therefore, it is thought that calprotectin chelates divalent metal ions that are required for microbial growth. This defense mechanism has been termed nutritional immunity [32]. Recently, Sroussi *et al.* proposed that the antifungal activity of calprotectin may be increased by oxidative stress [33].

Calprotectin is elevated in the extracellular fluids of patients with inflammatory disorders such as rheumatoid arthritis and vasculitis. Indeed, this complex is now used as a marker for inflammation [34]. Recently, calprotectin was described as a potential endogenous Toll-like receptor 4 (TLR-4) activator that promotes lethal endotoxic shock [35].

Despite all these important extracellular functions the complex lacks a secretion signal. A non-classical and tubulin-dependent secretion mechanism was shown in monocytes activated by inflammatory cytokines [36]. The mechanism by which this cytoplasmic dimer derived from neutrophils is able to interact with extracellular microbes *in vivo* is not completely understood. Here we show that calprotectin is released as the major antifungal protein in NETs. Our data indicate that at infection sites NET formation is a mechanism which ensures the interaction between cytoplasmic calprotectin and extracellular microbes at high local concentrations.

## Results

### Qualitative analysis of the protein composition of NETs

We isolated neutrophils from healthy donors and induced them to form NETs using phorbol myristate acetate (PMA). After gently washing the NETs twice to remove unbound proteins, we solubilized NET-bound proteins with DNase-1 (Figure S1). NET proteins were digested with trypsin and analyzed by nano-scale liquid chromatography coupled matrix-assisted laser desorption/ionization mass spectrometry (nano LC-MALDI-MS). The identification quality is represented by the MS/MS spectrum of S100A9, a subunit of calprotectin found with this approach (Figure S2 A). A protein was considered to be associated to NETs when the identification criteria (see Methods) were fulfilled in at least two from a total of three independent samples. We identified 24 different proteins (Table 1, NET Database: http://web.mpiib-berlin.mpg.de/cgi-bin/pdbs/lc/index.cgi), nine of which were previously shown to localize in NETs by immunofluorescence microscopy [3],[37], which correlates well with the results of our approach.

**Table 1:** Summary of identified NET proteins.

Cellular localization	Protein name	Gene name	Swissprot/TREMBL
**Granules**	Leukocyte elastase	*ELA2*	P08246
	Lactotransferrin	*LTF*	P02788
	Azurocidin	*AZU1*	P20160
	Cathepsin G	*CTSG*	P08311
	Myeloperoxidase	*MPO*	P05164
	Leukocyte proteinase 3	*PR3*	P24158
	Lysozyme C	*LYZ*	P61626
	Neutrophil defensin 1 and 3	*DEFA-1 and -3*	P59665, P59666
**Nucleus**	Histone H2A	*H2A*	Q9NV63+
	Histone H2B: a) Histone H2B	*H2B*	Q16778+
	b) Histone H2B-like	*H2B*	Q3KP43, Q6GMR5
	Histone H3	*H3*	Q71DI3+
	Histone H4	*H4*	P62805+
	Myeloid cell nuclear differentiation antigen	*MNDA*	P41218
**Cytoplasm**	S100 calcium-binding protein A8	*S100A8*	P05109
	S100 calcium binding protein A9	*S100A9*	P06702
	S100 calcium-binding protein A12	*S100A12*	P80511
**Cytoskeleton**	Actin ( 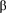 and/or 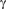 )	*ACTB*, *ACTG1*	P60709, P63261
	Myosin-9	*MYH-9*	P35579
	Alpha-actinin (1 and/or -4)	*ACTN1*, *ACTN4*	P12814, O43707
	Plastin-2	*LCP1*	P13796
	Cytokeratin-10	*KRT-10*	P13645
**Peroxisomal**	Catalase	*CAT*	P04040
**Glycolytic enzymes**	Alpha-enolase	*ENO1*	P06733+
	Transketolase	*TKT*	P29401

Proteins that localize to NETs. Proteins are organized by their localization in unstimulated neutrophils. Those identified to be NET-associated for the first time in this report are shown in blue. Swissprot/TREMBL accession numbers marked with a “+” denote possible forms of the protein that cannot be discriminated by this analysis. We identified two distanced groups of histone H2B. The larger group of individual histone H2B types we refer to as H2B and the other type that is less well characterized we refer to as “H2B-like”. All possible accession numbers can be found in the NET Database (http://web.mpiib-berlin.mpg.de/cgi-bin/pdbs/lc/index.cgi).

We identified proteins that have a nuclear, granular or cytoplasmic localization in unstimulated neutrophils. Among the nuclear components, we confirmed the presence of all four subtypes of core histones and newly found the myeloid cell differentiation antigen (MNDA). We also identified eight granular proteins, five of which (neutrophil elastase (NE), lactotransferrin (LTF), cathepsin G (CG), myeloperoxidase (MPO) [3] and more recently proteinase 3 (PR3) [37]) were previously found associated with NETs. Azurocidin, lysozyme C (LysC) and α-defensins were not known to be NET components. Notably, we found eleven cytoplasmic proteins; two glycolytic enzymes, catalase, five cytoskeletal proteins and three S100 proteins. We confirmed NET association of twelve proteins found in this study by indirect immunofluorescence. Notably, the linker histone H1, bactericidal/permeability increasing protein (BPI), pentraxin 3 (PTX-3) and cathelicidin (CAP-18), were described as NET-associated [3],[38],[39], but we did not find them with this approach. Immunoblot analysis, however, confirmed the presence of BPI, but not of PTX-3 or CAP-18 (Figure S2 B–D).

**Figure 1:**
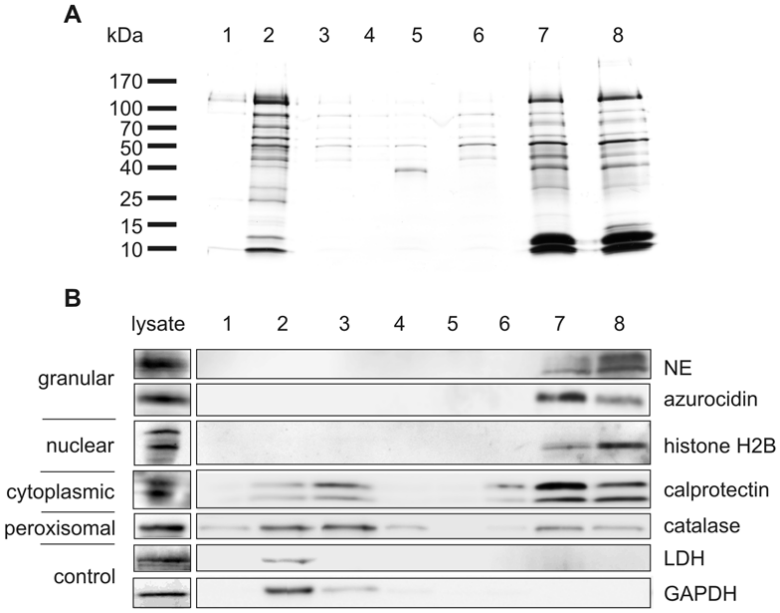
Identification of NET-associated proteins. (A) Silver stained SDS-PAGE and (B) immunoblots with samples from NET protein purification procedure. Human neutrophils were stimulated to form NETs. Supernatants from unstimulated (lane 1) and stimulated (lane 2) neutrophils; first wash (lane 3); second wash (lane 4); medium containing DNase-1 incubated with unstimulated neutrophils (lane 5); DNase-1-free medium incubated with washed NETs (lane 6); medium containing DNase-1 incubated with washed NETs (lane 7); medium containing DNase-1 incubated with washed NETs including protease inhibitor cocktail (lane 8).

To evaluate the specificity of our approach, we analyzed all purification steps. As previously described, activated neutrophils release many unbound proteins into the supernatant [40] (Figure 1A and B, lane 2) which were removed by two washes with culture medium (Figure 1A and B, lane 3 and 4). NET-associated proteins were specifically released by Dnase-1 (Figure 1A and B) with or without protease inhibitor cocktail (lane 7 and 8 respectively). As an additional control for the specificity of our approach we demonstrated that two cytoplasmic proteins, glyceraldehyde-3-phosphate dehydrogenase (GAPDH) and lactate dehydrogenase (LDH), were present in the supernatant (Figure 1B, lane 2), but not in the nuclease-digested NETs (Figure 1B, lanes 7 and 8).

To control the efficiency of the washing steps we increased the number of washes from two to nine (1 ml/wash, Text S1) in similar experiments as described above and determined the amount of calprotectin, a very abundant protein in neutrophils, in each fraction with ELISA. After NET formation calprotectin was present in similar amounts in culture supernatants and in wash 1 (Figure S2 E). The calprotectin concentration dropped in wash 2. These data agreed well with the immunoblot data presented in Figure 1B. However, we still detected minor amounts of calprotectin in wash 3 (55 ng/ml) and 4 (21 ng/ml). The amount decreased further in washes 5–7 until the calprotectin concentration reached the detection limit (1.6 ng/ml) of the assay in wash 8 and 9. Notably, from digestion of these intensely washed NETs with 1 ml RPMI containing 5 U/ml MNase we obtained approximately 170 ng/ml calprotectin which equals 23% of the total amount (720 ng/ml) according to our determination with the same ELISA under the same conditions. This is 20-fold more calprotectin than in wash 7 suggesting that calprotectin is bound to NETs. Taken together, this indicates that we purified and selectively enriched NET-associated proteins and that the NETs contain at least 24 proteins 15 of which were identified as NETs component for the first time (Table 1, marked in blue, and NET Database).

### Quantitative analysis of the protein composition of NETs

To determine the relative amounts of NET proteins we quantified 15 of the 24 NET-associated proteins by immunoblotting (Table 2, Figure S3, NET Database). We purified NET proteins from neutrophils isolated from ten healthy donors. On average, NETs derived from 10^12^ neutrophils contained 3.58+/−0.28 g of protein and 2.24+/−0.51 g of DNA. This indicates a ratio of 1.67+/−0.26 g of protein per gram of DNA.

**Table 2:** Quantified NET proteins.

Protein name	Molecular weight (KDa)	µmoles (per g NET-DNA)	% Molar amount	mg protein (per g NET-DNA)
Histone H2A	16.1	23.60±1.07	26.29	379.3±17.3
Histone H2B	15.3	21.50±0.77	23.95	298.9±10.7
Histone H3	13.9	13.02±1.39	14.50	199.2±21.3
Neutrophil elastase	25.4	5.24±0.85	5.84	133.0±21.5
Histone H4	11.4	3.96±0.39	4.41	45.2±4.5
S100A8	10.8	3.59±0.48	4.00	38.8±5.2
Lactotransferrin	76.0	2.46±0.28	2.74	186.6±21.6
Azurocidin	23.8	2.35±0.44	2.62	55.9±10.6
Cathepsin G	26.6	2.22±0,11	2.47	59.2±2.7
S100A9	13.2	1.27±0.12	1.41	16.7±1.6
Myeloperoxidase	78.4	0.91±0.07	1.01	71.3±5.3
Proteinase 3	24.0	0.64±0.13	0.71	15.5±3.2
Actin	41.8	0.15±0.02	0.17	6.3±0.9
Lysozyme C	14.5	0.12±0.036	0.13	1.8±0.5
Catalase	59.8	0.02±0.001	0.02	1.25±0.07

Quantified NET proteins are listed according to their µmolar amounts normalized to the amount of NET-DNA. Additionally, the calculated amount as a percentage of total NET protein content and the mass per g NET-DNA are shown. The data are representative mean values±mean deviation (n = 3) from two independent experiments.

**Figure 2:**
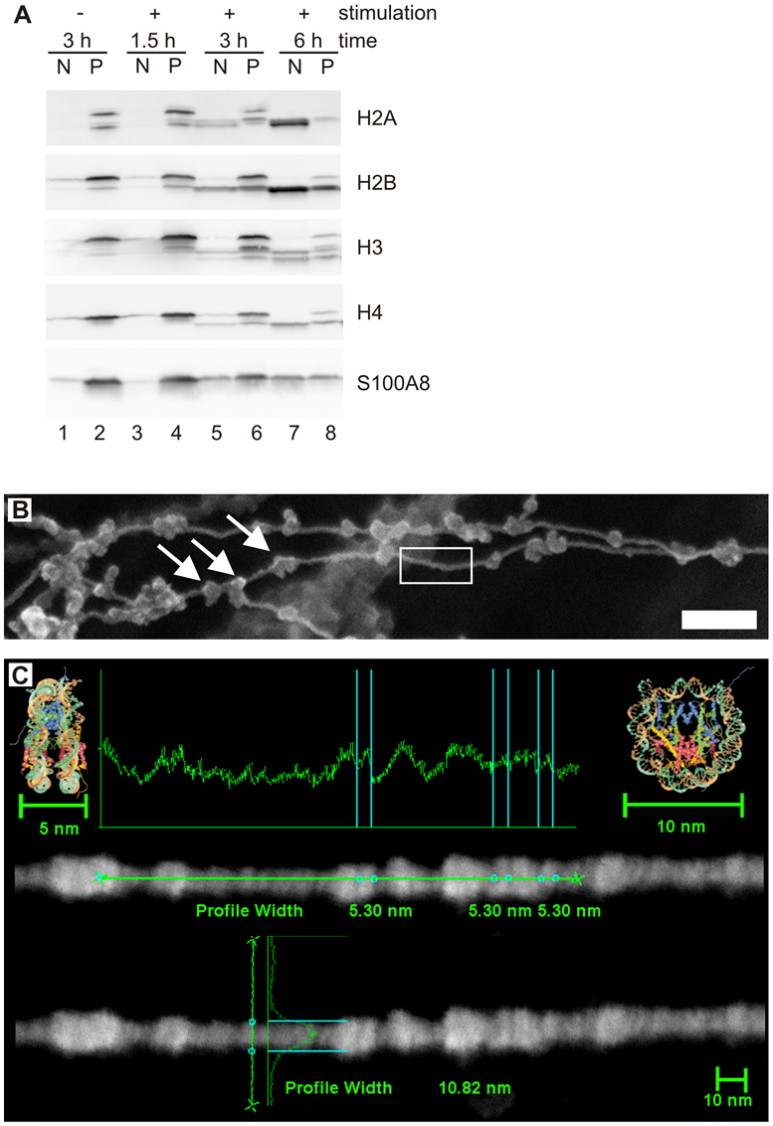
Histones are altered during NET formation. NETs from human neutrophils were washed and digested with DNase-1. (A) The NET-fraction (N) and the remaining pellet after DNase-1 digest (P) were analyzed by immunoblotting at the indicated time points. Unstimulated neutrophils served as controls. All core histones have a reduced molecular mass (2–5 kDa less) in NETs compared to the pellet fraction and the unstimulated control. A representative experiment out of three in total is shown. (B) High-resolution SEM analysis of NETs which consist of smooth fibers (white box) and globular domains (diameter 25–50 nm, arrows), scale bar = 100 nm. (C) High-resolution FESEM analysis of smooth stretch of a singular NET-fiber. Signal intensities were profiled vertically and horizontally showing similar diameters to nucleosomes (depicted as cartoon structure models taken from [41], with approximate horizontal and vertical diameters of 5 nm and 10 nm, respectively). One experiment out of two is shown.

The core histones H2A, H2B, H3 and H4, were the most abundant proteins and account for 70% of all NET-associated proteins. The molecular mass of these proteins is decreased by approximately 2–5 kDa, when compared to histones present in the nucleus (Figure 2A). This modification is specific, since the masses of non-nuclear proteins do not change upon association with NETs. (Figure 2A, S100A8 and NET Database). Moreover, the stoichiometry of the four core histones is different in NETs as compared to intact nuclei. In unstimulated neutrophils, the core histones are present in similar amounts (Figure 2A, lane 2). In contrast, on NETs, H3 and H4 are found in lower amounts than H2A and H2B (Figure 2A, lane 7) and their molarity per gram NET-derived DNA is different (Table 2, NET Database). These observations correlated to high resolution Field Emission Scanning Electron Microscope (FESEM) analyses (Figure 2B and C). NETs consist of “smooth” stretches, probably only composed of histones and DNA (Figure 2B, white box), interspersed with globular domains that contain granular proteins [3]. The smooth stretches showed periodical signal intensities that are approximately 5 nm thick and 10 nm wide (Figure 2C). These dimensions are similar to those of nucleosomes [41] suggesting that smooth NET stretches are composed of stacked cylindrical nucleosomes. On different areas we found intensities with a similar horizontal periodicity but a lower vertical dimension consistent with the notion that the histone composition was changed during NET formation (Figure 2A).

The most abundant non-histone NET protein was NE and the least abundant protein detected was catalase (5.24 and 0.02% of total protein, respectively). The 15 proteins we quantified represent 90.3% of the total protein in NETs. Thus, the remaining nine proteins and those that were potentially not identified in our approach represent 9.7% of the total NET proteins. NETs contained 39 ng of S100A8 and 17 ng of S100A9 per µg of DNA after 2 washes (Table 2 and NET database). Since each well yielded in average 3.8 µg DNA, we calculated 148 ng S100A8 and 65 ng S100A9 per well. Both proteins add up to a total of 213 ng/well. This correlates well with the quantification of calprotectin by ELISA (Figure S4 E) which yielded 170 ng/well. It is possible that the difference observed is due to the different number of washes in the two experiments.

Taken together, the different quantification methods are comparable.

### Calprotectin localizes in NETs

**Figure 3:**
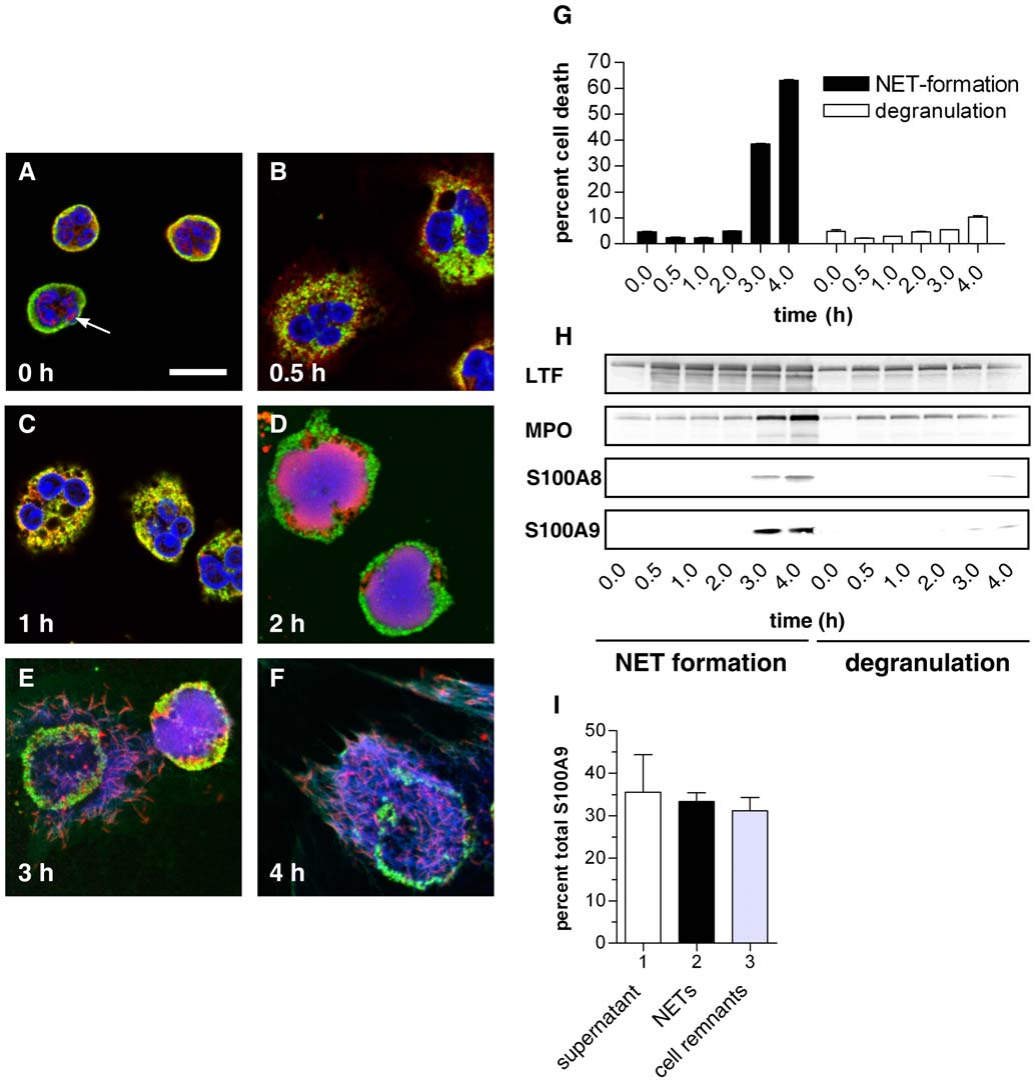
Neutrophils release calprotectin by forming NETs. (A–F) Confocal images of human neutrophils without stimulation (A), after 0.5 h (B), 1 h (C), 2 h (D), 3 h (E) and 4 h (F) after activation. Samples were stained with antibodies specific for the calprotectin heteroduplex (red) and for MPO (green). DNA was stained with DRAQ5 (blue). Calprotectin localizes to the cytoplasm and partially to the nucleus (A, arrow). After stimulation for 0.5 h (B) the neutrophils flattened and formed numerous vacuoles. This reveals a granular staining for MPO and a more dispersed cytoplasmic staining for calprotectin. After stimulation for 1 h (C) the neutrophils round up slightly. The MPO and calprotectin stain partially overlap in the cytoplasm. After stimulation for 2 h (D), calprotectin, MPO and nuclear DNA start to colocalize in the decondensed nucleus (purple). After 3 h (E) and more so after 4 h (F) of stimulation, the cell membrane ruptures and calprotectin is released in NETs colocalizing with MPO and DNA. Scale bar = 10 µm; one experiment out of two is shown. (G–I) Subunits of calprotectin S100A8 and S100A9 are released after cell death during NET formation and not by degranulation. NET formation was induced with PMA and degranulation using formyl-met-leu-phe (f-MLP). (G) Neutrophil death was monitored by quantification of LDH activity in supernatants calculated as means±s.d. (n = 3). (H) Release of S100A8, S100A9, lactotransferrin (LTF) and myeloperoxidase (MPO) were analyzed by immunoblotting. one experiment out of two is shown. (I) Quantification of immunoblots using 2D densitometry analyzing S100A9 protein preparations from supernatants (lane 1), MNase-digested NETs (lane 2) and cell remnants indigestible for MNase (lane 3). Values were calculated as means±s.d. (n = 3) from one experiment out of two.

The association of calprotectin to NETs suggests that the complex is released during a specific form of holocrine secretion [42] referred to as NETosis [43]. Using indirect immunofluorescence we verified that calprotectin is released through NET formation *in vitro* (Figure 3A–F). A calprotectin-heteroduplex-specific antibody [44] confirmed that the dimer was cytoplasmic (red), and it overlapped with granular MPO (green) in the compact cytoplasm of unstimulated cells (Figure 3A). There was also a faint calprotectin signal within the nucleus (DNA stain, blue) consistent with the fact that cytoplasmic proteins with a mass below 30 kDa can diffuse through the nuclear pore complex. Thirty minutes after stimulation the neutrophils flattened as a sign of activation revealing a granular staining for MPO and a more dispersed cytoplasmic staining for calprotectin (Figure 3B). After stimulation for 1 hour, we observed partial colocalization of MPO and calprotectin in the cytoplasm (Figure 3C). This increased two hours after activation, when the chromatin decondensed and the nuclear membrane disassembled. At this time point calprotectin, MPO and DNA colocalized (Figure 3D). A proportion of the MPO signal remained in the area between the plasma membrane and the decondensed nucleus. Three and 4 hours post-activation, when the plasma membrane ruptured, calprotectin, DNA and MPO colocalized on NETs (Figure 3E–F).

To determine whether calprotectin is released during NET cell death or before the plasma membrane ruptures, we compared neutrophils that were treated either with PMA to stimulate NET formation [3] or with the microbial peptide formyl-met-leu-phe (f-MLP) to stimulate degranulation [40]. At the indicated time points we monitored neutrophil cell death by quantifying extracellular LDH (Figure 3G) and solubilized NET-bound proteins by adding Dnase-1 (Figure 3H). We subsequently collected the supernatants containing both unbound and NET-bound proteins to determine the total release of the indicated proteins during degranulation and NET formation. We analyzed the samples by immunoblotting and probed for the calprotectin subunits S100A8 and S100A9 as well as for two granular proteins, MPO and LTF (Figure 3H). Thirty minutes after f-MLP treatment, neutrophils released MPO and LTF, but not S100A8 nor S100A9. Four hours after f-MLP treatment, minor amounts of S100A8 and S100A9 appear in the supernatants and less than 10% of the cells were dead. During NET formation, MPO and LTF were secreted in low amounts before S100A8 and S100A9 were released confirming that neutrophils also degranulate upon PMA-activation [40]. Significant amounts of extracellular calprotectin were only found 3 to 4 hours after stimulation, when 40 and 70% of the neutrophils respectively were dead. Furthermore, we determined how much of the total calprotectin amount present in the neutrophil actually binds to NETs. After stimulation we quantified S100A9 released into the supernatant, bound to NETs and remaining in the cell remnants by quantitative immunoblots. Approximately 30% of the total S100A9 was bound to NETs (Figure 3I). Equivalent amounts of S100A9 were found in the supernatants and in the nuclease-resistant cell remnants (Figure 3I). This agrees with a previous report showing that after nuclease treatment, a proportion of chromatin remains within the debris of the neutrophil [6]. We obtained similar results for S100A8 (data not shown). Moreover, a calprotectin specific ELISA (Figure S2 E) confirmed the finding that similar amounts of calprotectin are in NETs (170 ng/ml) compared to unbound in the culture supernatant (200 ng/ml) and that this corresponds to 23% and 28% of the total amount of calprotectin respectively. Taken together, this demonstrates that approximately one third of calprotectin *in vitro* is released from neutrophils in NETs.

### Calprotectin is a major antifungal component of NETs

As we have reported previously NETs can control the growth of *C. albicans* yeast and hyphal forms [13] and more recently this has been demonstrated for *Aspergillus nidulans* conidia and hyphae as well [45]. Additionally, we have now added *Cryptococcus neoformans* to this list of NET inhibited fungi (Figure S4 A). However, the mechanism behind the antifungal activity remained unclear. In this study we confirmed the previous results by exposing *C. albicans* to NETs at different multiplicities of infection (MOI) and at different temperatures to induce yeast-form and hyphal growth (Figure S4 B and C). Under all conditions the antifungal activity of NETs was very similar reducing CFU counts approximately 100-fold, but not when the NETs were degraded with nucleases (Figure S4 D). We confirmed the antifungal activity of NETs against hyphae macro- and microscopically (Figure S4 E and F). As expected, hyphae grew in media but this growth was inhibited when they were incubated with NETs. We corroborated the anti-candidal activity of NETs and showed similar results measuring the viability of *C. albicans* hyphae with the tetrazolium dye XTT (Figure S4 G–I). This method, unlike CFU enumeration, does not require the dispersion of the hyphae [46].

We confirmed that NETs made by neutrophils stimulated with *C. albicans* contain calprotectin, lactotransferrin and catalase (Figure S4 J). This suggests that NETs have a qualitatively similar composition regardless of the stimulus used to activate the neutrophil.

**Figure 4:**
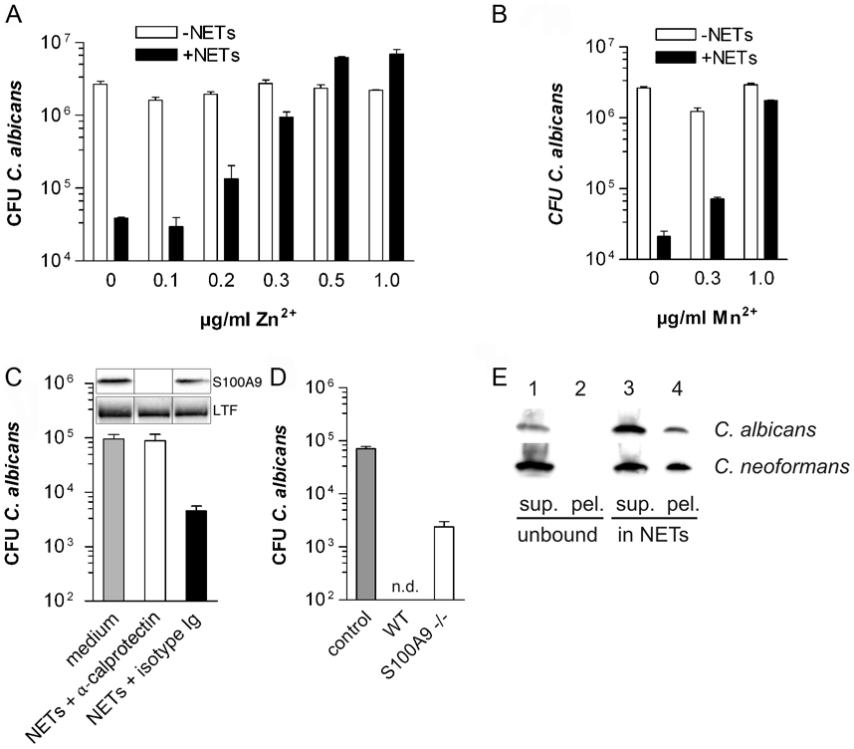
Calprotectin is a major antifungal component in NETs. Human neutrophils were induced to make NETs, washed and infected with *C. albicans* (MOI 0.01) and incubated overnight at 30°C (yeast-form growth). Antifungal activity was determined by counting CFU. Increasing concentrations of ZnSO_4_ (A) and of MnCl_2_ (B) abolished the antifungal activity of human NETs. Shown are means±s.d. (n = 3) from one representative experiment out of three. (C) Purified human NET proteins were concentrated and depleted of calprotectin using immobilized anti-S100A8 and anti-S100A9 combined. Controls were incubated with mouse IgG1 isotype matched antibodies. *C. albicans* was incubated with these extracts overnight at 37°C (hyphal growth) and CFU were determined. Shown are means±s.d. (n = 3) from one experiment out of two. The inset shows an immunoblot confirmation of the depletion. The blots were probed for S100A9 and lactotransferrin (LTF). The lanes are arranged in the same order as indicated for the antifungal assay below the graph. (D) Mouse neutrophils isolated either from wild-type or calprotectin-deficient mice were induced to make NETs and then infected with *C. albicans* (MOI 0.02) incubated overnight at 37°C (hyphal growth) and CFU counts were determined; n. d. = no detectable CFU. Shown are means±s.d. (n = 3) from one experiment out of two. (E) Co-precipitation assays of indicated fungi incubated with NET-bound calprotectin (produced by MNase treatment) or soluble calprotectin. Microbes were pelleted, supernatants removed and washed pellets were analyzed using immunoblots against S100A9. Shown are means±s.d. (n = 3) of one representative experiment out of three.

Calprotectin chelates essential metal ions, such as Zn^2+^ and Mn^2+^ resulting in reduced microbial growth [30],[31]. Consistent with this mechanism of action the antifungal activity of NETs was inhibited by increasing concentrations of Zn^2+^ (Figure 4A) or Mn^2+^ (Figure 4B). The direct role of calprotectin in NETs was tested with immunodepletion experiments. We purified NET proteins by DNase-1 treatment and concentrated the samples 10-fold with 3.5 kDa cut-off membranes. The samples were incubated with a mixture of immobilized antibodies directed against the individual subunits S100A8 and S100A9 (Figure 4C). Immunodepletion of calprotectin, but not treatment of NET preparations with isotype-matched controls, completely abrogated the growth-inhibitory activity of NET proteins showing that this dimer is a major antifungal component of NETs. It is important to note that immunoblotting of the supernatant showed that S100A9, but not lactotransferrin, was depleted (Figure 4C). A complementary assay where *C. albicans* viability was measured using XTT confirmed the result of the CFU based assay (Figure S4 H). Furthermore, NETs made by calprotectin-deficient mice inhibited *C. albicans* growth less efficiently than wild-type mouse NETs (Figure 4D) which was confirmed with an XTT assay as well (Figure S4 I). Notably, neutrophils from both genotypes made NETs with similar efficiency (Figure S5 A–F).

The antimicrobial activity of calprotectin does not require direct contact between microbe and protein, which is consistent with its inability to bind microbial surfaces [30],[31]. Regardless, at high concentration this chelator is more efficient. Therefore, we tested whether NET-bound calprotectin binds to microbes. Seeded neutrophils were induced to release NETs. We collected the supernatant after NET formation that contained unbound and soluble calprotectin. The NETs were subsequently washed twice and digested with MNase, a non-processive nuclease, to generate NET fragments. We exposed the indicated fungi to these NET fragments or supernatants containing soluble calprotectin and pelleted the microbes afterwards. The microbial pellets were washed three times. Immunoblotting in Figure 4E shows that NET-associated, but not soluble S100A9, binds to the fungi. We obtained similar results for S100A8 (data not shown). Taken together, these data indicate that calprotectin is a major antifungal NET component. We conclude that the presentation of this dimer in NETs provides a high local concentration on the surface of microbes.

### Calprotectin is required for innate antifungal defense

We addressed the role of calprotectin in *C. albicans* infections comparing wild-type to *S100A9* knockout mice. These animals transcribe the mRNA for S100A8 but are deficient in S100A8 and S100A9 protein [47]. *C. albicans* exploits different host niches and we investigated three of them: (i) subcutaneous inoculation, which leads to confined abscesses; (ii) intranasal infection, which causes pulmonary candidiasis and (iii) intravenous challenge, which mimics disseminated systemic candidiasis.

**Figure 5:**
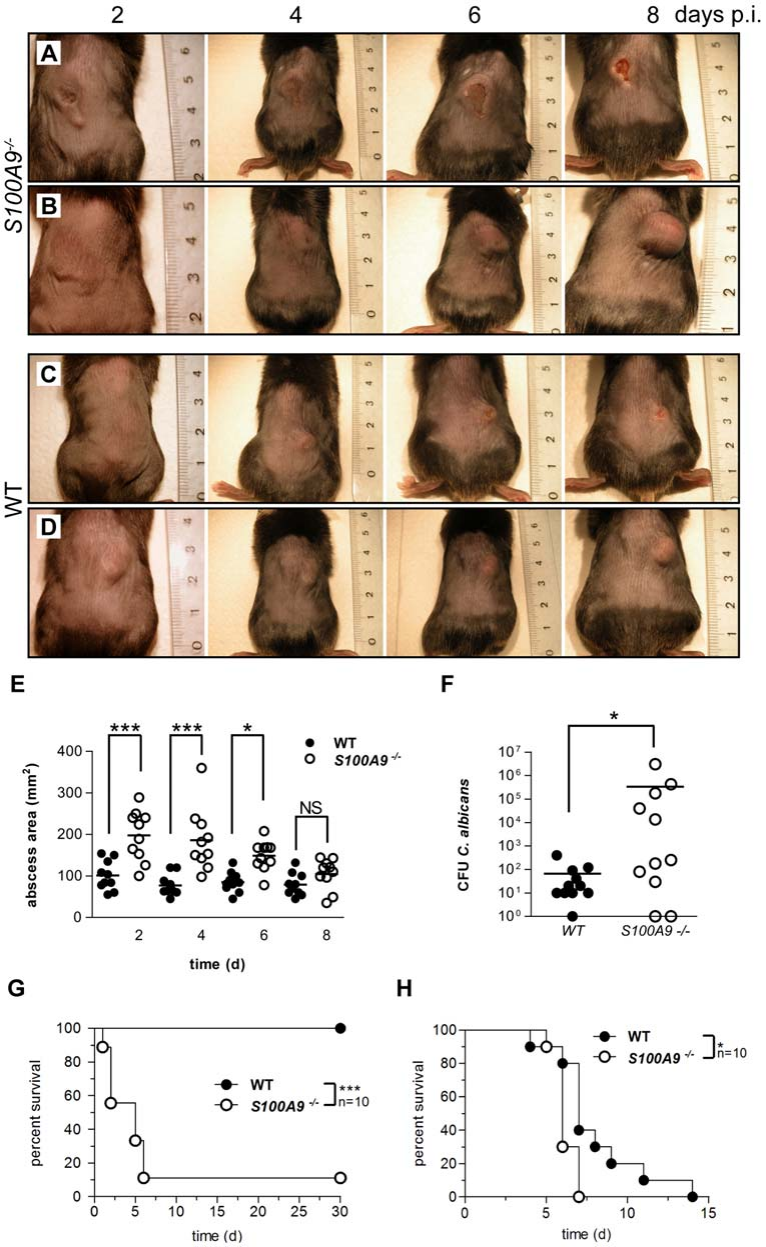
Calprotectin is required for antifungal immunity. (A–D) Subcutaneous abscesses induced with *C. albicans* of representative calprotectin -deficient (a and b) and wild-type (c and d) mice at days 2, 4, 6 and 8 post infection (p.i.). (E) the area of the abscess lesions of calprotectin-deficient animals compared to wild type was monitored over 8 days p.i. (n = 10); abscesses of calprotectin-deficient animals are significantly larger at days 2 (P = 0.0007), 4 (P = 0.0012), 6 (P = 0.0003) but not at day 8. (F) calprotectin-deficient mice are more susceptible to intranasal challenge with *C. albicans* than wild-type mice (n = 10, P = 0.0001). (G) Fungal load in the lungs after intranasal challenge with *C. albicans* is significantly higher in calprotectin-deficient compared to wild-type mice (n = 11; P = 0.048). (H) Calprotectin-deficient mice are more susceptible to intravenous challenge with *C. albicans* than wild-type mice (n = 10, P = 0.0159).

The dimensions of the abscess lesions were measured at indicated time points. On average, the area of the lesions in calprotectin –deficient mice were twice as large (200 mm^2^) as compared to those of wild-type animals (100 mm^2^) measured at two, four and six days after inoculation (Figure 5A–E). At day four, 60% of the abscesses in calprotectin-deficient mice ulcerated as indicated by extensive necrosis which also included the epidermis (Figure 5A). Consistent with a less severe progression, abscesses of wild-type animals were restricted to subcutaneous areas without involvement of outer skin layers (Figure 5D). Eight days after inoculation, the size of the abscesses in calprotectin-deficient and wild-type mice was similar, indicating that calprotectin is essential for the initial phase of the disease but that eventually other mechanisms clear the infection. Interestingly, in about 30% of the infected knockout mice, but not in wild-type controls, infection spread from the original abscess to surrounding areas of the skin (Figure S6). In our experimental settings neutrophil recruitment (Figure S5 G) and NET formation (Figure S5 A–F) was similar in knockout and wild-type animals.

In pulmonary candidiasis, wild-type mice show disease symptoms within three days but recover and survive. In contrast, calprotectin-deficient mice carried a significantly higher fungal load than wild-type controls (Figure 5F) and succumbed to *C. albicans* (Figure 5G).

Intravenous challenge is lethal in both calprotectin-deficient and wild-type mice. However, the knockout animals died significantly earlier (Figure 5H), suggesting that also in deep-seeded infection sites, the antimicrobial activity of calprotectin contributes to containment of fungal growth. This is consistent with a previous report that calprotectin reduces bacterial load in systemic staphylococcal infection [31]. We conclude that calprotectin is required for an effective acute antifungal response.

### NETs released during *C. albicans* infection contain calprotectin

**Figure 6:**
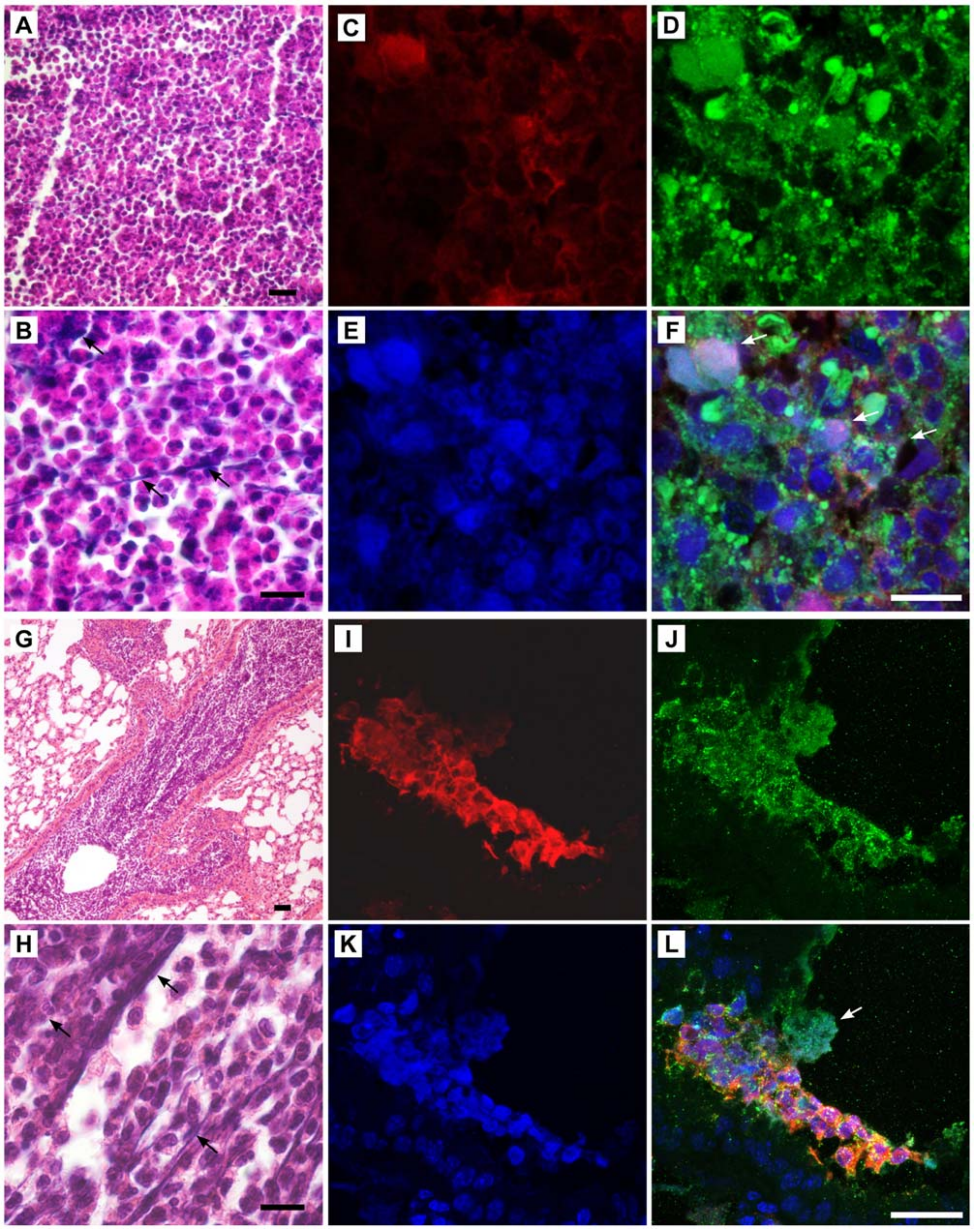
Calprotectin is present in *C. albicans* induced NETs *in vivo*. Wild-type mice were challenged subcutaneously (A–F) or intranasally (G–L). (A,B, G and H) Hematoxylin & Eosin (H & E) stainings of sections in areas with strong neutrophil infiltration show extracellular DNA (hematoxylin positive) representing NETs and indicated with arrows, scale bars 50 µm in (A,G) and 20 µm in (B,H). Confocal images of indirect immunofluorescence from sections of abscesses 6 days after subcutaneous challenge (C–F) and lungs 24 h after intranasal challenge (I–L) stained with primary antibodies against S100A9 (red), MPO (green) and histone (blue). NETs are web-like and diffuse areas where all signals superimpose indicated by white arrows. Scale bars = 20 µm.

We tested whether NET formation is a route of calprotectin release *in vivo* during subcutaneous (Figure 6A–F) and pulmonary *C. albicans* infection (Figure 6G–M). Histological analysis of 6 day old abscesses showed fungal foci surrounded by neutrophils (Figure 6A). Using staining with hematoxylin and eosin (H &E), we observed abundant extracellular DNA in web-like structures in these areas (Figure 6B). To confirm that these structures were NETs, the samples were labeled with antibodies directed against MPO (green) and histones (blue) and analyzed by immunofluorescence microscopy. Labeling with anti-S100A9 antibodies demonstrated that these structures also contain calprotectin (Figure 6, C–F).

**Figure 7:**
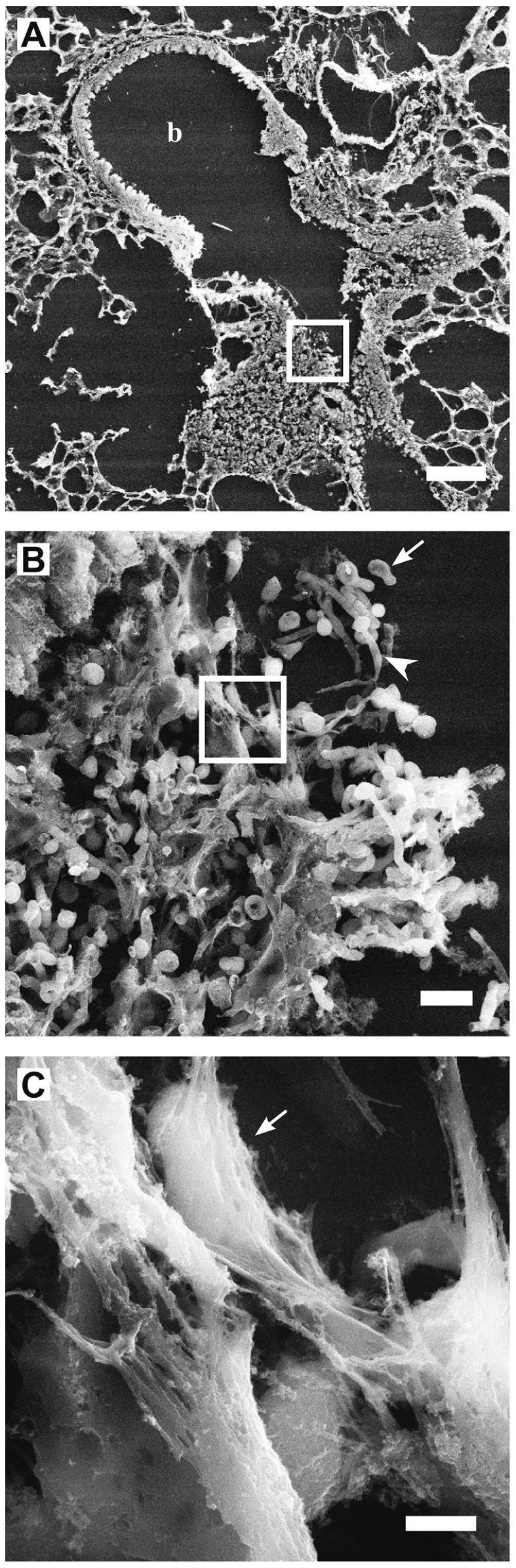
Fine structure of *C. albicans* induced NETs in pulmonary infection. (A–C) SEM analysis of sections from *C. albicans* infected mouse lungs 24 h after intranasal challenge. (A) Image shows a bronchiole colonized with *C. albicans* and infiltrated by host immune cells, b = bronchiole. (B) High resolution image of boxed area in (n) shows respiratory epithelium of the bronchiole colonized with *C. albicans* yeast-form (arrow) and hyphae (arrowhead). (C) High resolution image of boxed area in (O) showing NETs covering fungal surfaces (arrow). Scale bars in (N) = 100 µm, in (O) = 10 µm and in (P) = 2 µm.

In infected lungs, there was a strong neutrophil infiltration into *C. albicans* colonized bronchioles one day after challenge. These areas showed web-like structures of extracellular DNA (Figure 6 G and H). MPO and histones (Figure 6M, arrow), as well as calprotectin(Figure 6I–l) colocalized in these structures that unfold into the lumen of the bronchioles. To demonstrate that *C. albicans* and NETs interact in the lumen of bronchioles we analyzed these sections by Scanning Electron Microscopy (SEM) (Figure 7A). Areas colonized by *C. albicans* show web-like structures that cover fungal surfaces (Figure 7B and C). These structures have a very similar morphology and dimension to those observed for NETs *in vitro*. Taken together these observations demonstrate that NETs and *C. albicans* interact *in vivo* and that these NETs contain calprotectin. We propose that release and NET-association of calprotectin from neutrophils could contribute to contain fungal infections.

## Discussion

The analysis presented here identified 24 NET-associated proteins. Nine of the 24 proteins were described previously as NET-associated which correlates well to our approach. Fifteen proteins were hitherto unknown to be NET-bound and their association to NETs was confirmed by immunoblotting and indirect immunofluorescence (NET Database). The data underscore the specificity and reproducibility of the analysis. Moreover, the composition of the identified NET proteins was very similar among different neutrophil donors (NET Database).

The small number of identified NET proteins is surprising since the neutrophil cell membrane ruptures during the process of NET release. Additionally, we used mild washing conditions for the isolation. Therefore, the protein incorporation into NETs appears to be selective. We found that, in addition to five nuclear proteins, the NETs contain eight cationic granule proteins. Furthermore, we identified eleven cytoplasmic proteins which have neutral to acidic isoelectric points. This suggests that charge is not an exclusive requirement for binding to NETs.

In previous reports histone H1 [3], bactericidal/permeability increasing protein (BPI) [3], cathelicidin (CAP-18) [37],[39] and pentraxin 3 (PTX-3) [38] have been described as NET-associated proteins. In contrast, we did not find these proteins in our MS approach. We further investigated these proteins by immunoblot (Figure S2B–C). We did not obtain clear results for histone H1 because commercially available anti H1-antibodies are cross-reactive with other histones (data not shown). This remains to be clarified. We could, however, detect the 25 kDa cleavage product of BPI in both neutrophil lysates and NET extracts. The failure of MS analysis to detect BPI remains unclear (Figure S1B). Notably, we detected CAP-18 and PTX-3 in neutrophil lysates but not in NET extracts. Therefore the presence of these two proteins in NETs should be further investigated. It is possible that these proteins are very loosely attached to NETs, that they are present in very low amounts, that they were lost during the isolation procedure or that the MS analysis failed to detect them. Notably, neither by immunoblotting nor by MS did we find GAPDH, a very abundant cytoplasmic and highly cationic protein. This supports the assumption that NET binding is not exclusively mediated by charge.

Quantification by immunoblots showed that 15 NET proteins comprised 90% of the total protein amount in NETs, indicating good coverage. Detection of 1.25 ng of catalase per µg NET-DNA underscored the sensitivity of the approach. As the amounts of NET proteins were similar in different donors the approach was also reproducible (NET Database). More importantly this suggests that NETs do not assemble randomly and are similar in many individuals.

The core histones are the major protein components in NETs as shown by the quantitative analysis. However, we found a reduction of the molecular mass of NET-associated compared to chromatin histones, possibly caused by post-translational modifications. Indeed, histone H3 is deiminated during NET formation in HL60 cells but this remains to be confirmed in primary neutrophils [48]. Additionally, the stoichiometry of the four histones is different in NETs when compared to chromatin. There is less H3 and less H4 in NETs than in chromatin, but the significance of these differences remain unclear. High resolution FESEM correlated with this finding. We determined a transverse periodicity in individual NET-fibers that was similar to the actual dimensions of intact and partially degraded nucleosomes.

The role of some of the identified cytoplasmic proteins in NETs is unknown but there are indications for potential functions. Enolase, for example, has been found to be a plasminogen activator on leukocyte surfaces, although the secretion mechanism is unknown [49]. NET-association could explain how enolase is released allowing it to participate in tissue remodeling at inflammatory sites.

We identified S100A8 and S100A9 that form the heterodimer calprotectin as NET-associated proteins by MS analysis and by indirect immunofluorescence. The dimer lacks a secretion signal and localizes to the cytoplasm as well as partially to the nucleus of unstimulated neutrophils. Therefore, the proteins must be released in order to function as an antimicrobial protein against extracellular pathogens. The release of calprotectin by neutrophils [50] and other cells [36] in response to specific stimuli was previously reported. Our data, however, establish NET formation as a hitherto unrecognized mechanism of calprotectin release in neutrophils. Interestingly, incubation of *C. albicans* with neutrophils was reported to increase extracellular calprotectin and, simultaneously decrease neutrophil viability [51]. The release mechanism, however, remained unknown. Consistent with these data we reported that *C. albicans* induces neutrophils to form NETs and die in the process [13],[52]. Here, we demonstrate that NET formation induced by *C. albicans* during infection is a novel route for release and presentation of calprotectin *in vivo*. We additionally showed that calprotectin tightly binds to NETs as several intense washes cannot remove calprotectin from NETs (Figure S2 E) or NET-bound calprotectin from fungal surfaces (Figure 4E). In contrast the unbound proportion of calprotectin released during NET formation does not adhere to fungal surfaces. These findings suggest that calprotectin, although it is abundant in unstimulated neutrophils, specifically interacts with NETs. We determined that 30 and 23% of the total calprotectin was NET-associated by immunoblot densitometry (Figure 3I) and ELISA (Figure S2E respectively). These values indicate that a significant proportion of the protein complex associates with NETs. Additionally, approximately the same amount of calprotectin is released to the supernatant. Thus, probably 50–60% of total cellular calprotectin might become extracellular during NET formation. It is important to note that with other methods previous publications determined the total amount of calprotectin to be approximately 5 µg/10^6^ human neutrophils [53],[54]. Here we report approximately a ten-fold lower amount of calprotectin (0.43 µg/10^6^). This difference could be due to the methodology, donor variation and the technique to count cells. Our functional assays on the inhibition of *C. albicans* growth by NETs suggest that calprotectin in NETs might have a biological implication due to the high local concentration and close contact to the microbe.

In agreement with the findings discussed above we showed that calprotectin is a major antifungal protein in NETs affecting *C. albicans* and *C. neoformans*. Addition of Zn^2+^ or Mn^2+^ and immunodepletion of calprotectin abrogated the antifungal activity of NETs. This is consistent with previous reports showing that calprotectin is microbiostatic *in vitro* because it chelates ions [24],[25],[26],[30]. Notably, an enhancement of calprotectin's antifungal activity by oxidative stress has been reported recently [33]. This could as well be the case in NETs, since the release requires a robust oxidative burst to occur before [6]. Other NET proteins were not affected by calprotectin depletion (Figure 4C), but nonetheless NETs contain more antifungal proteins, such as azurocidin and LTF. Interestingly, addition of Fe^2+^ did not have an impact on *C. albicans* in our experimental settings (data not shown), suggesting that calprotectin, rather than LTF, controls *C. albicans* under these conditions. This is probably also true for *C. neoformans*. Fractionations of neutrophil compartments revealed previously that azurophilic granule extract inhibited cryptococcal growth to 80% and cytoplasmic extract to 70% [55]. The group identified calprotectin as the one single effector in the cytoplasm responsible for the strong inhibition correlating well with our findings. Whether other proteins contribute to the antifungal activity of NETs still remains to be determined (Figure 4D). Moreover, our data demonstrate for the first time that calprotectin is required for the innate immune defense to *C. albicans* infections. We detected abundant NETs in subcutaneous abscesses (Figure 6A–F), lungs after pulmonary challenge (Figure 6 G–M) and kidneys from systemic candidiasis (data not shown), and we propose that they prevent the spread of *C. albicans*. This might be in particular important for *C. albicans* hyphae that are too large to be engulfed and is consistent with our previous finding that hyphae are more potent NET inducers as yeasts [52]. Thus, we conclude that NET formation could serve as an additional neutrophil-mediated anti-hyphal mechanism complementing the previously described damage of hyphae by oxidative products and the myeloperoxidase system [56],[57]. The NET-released calprotectin reduces *C. albicans* growth correlating well with the more severe disease symptoms we observed in calprotectin-deficient mice as compared to wild type: increased abscess sizes upon subcutaneous challenge and higher fungal loads in lungs upon intranasal challenge. Indeed, the importance of NETs in reducing dissemination seems likely to be similar to that observed in streptococcal pneumonia [10]. This is consistent with a stronger survival phenotype of calprotectin in pulmonary compared to systemic infection, where *C. albicans* was disseminated from the outset. We and others determined neutrophils as a major source of calprotectin in infected tissue under the given conditions (data not shown, [31]). However, calprotectin has been reported to be expressed by other myeloid and epithelial cells and to be involved in numerous other immune-related functions, such as chemotaxis, regulation of the NADPH oxidase complex, activation of Toll-like receptor 4 and induction of apoptosis [35],[58],[59],[60]. Any of these functions may contribute to the phenotypes in *C. albicans* infection we report here. So far we have not identified a component that is exclusively required for NET formation but not other neutrophil functions. Therefore, we do not have a tool to determine the actual contribution of NET-associated calprotectin in the described infection phenotypes. We propose that differential localization of calprotectin, *e. g.* unbound and NET-bound, may enable the protein to be multi-functional.

Our analysis revealed a simple, stable and reproducible composition of NETs. These data are likely to help investigate the functions of NETs in infections and in other disorders such as autoimmune diseases.

## Materials and Methods

### Mice, cells and strains

*S100A9* ^−/−^ mice [47] that are deficient in both calprotectin subunits S100A8 and S100A9 protein were backcrossed 7 times into C57 BL/6. These mice and C57 BL/6 controls were bred in our animal facility. All animal experiments were in compliance with the German animal protection law in a protocol approved by the Landesamt fur Gesundheit und Soziales, Berlin. Human peripheral blood neutrophils and mouse bone marrow derived neutrophils were isolated as described [6],[61]. We used *C. albicans* clinical isolate SC 5314 [62]. *C. neoformans* strain DSM 11959 was obtained from the German Collection of Microorganisms and Cell Cultures (DSMZ). *C. albicans* was cultured overnight in YPD (1% yeast extract, 2% bacto peptone and 2% glucose) at 30°C and *C. neoformans* in the same medium at 37°C. Cells were washed 3 times in PBS prior to the experiments. Cell numbers were calculated by OD_600_ correlation (*C. albicans*: 1 OD_600_ = 3×10^7^ cells/ml; *C. neoformans*: 1 OD_600_ = 6×10^7^ cells/ml). For experiments with neutrophils and NETs *C. albicans* was either incubated at 37°C to induce hyphae or at 30°C to preserve yeast-form growth. *C. neoformans* was always incubated at 37°C. All infection assays were performed in RPMI medium.

### Purification of NET proteins

Human neutrophils were seeded in 12-well tissue culture plates to a density of 1.7×10^6^ ml^−1^ (RPMI 1640 without phenol red). 1.7×10^6^–3.4×10^6^ (according to 1–2 wells) and 1.7×10^7^ (according to 10 wells) neutrophils were used for analytical and MS identification respectively. Neutrophils were activated with 20 nM PMA for 4 h at 37°C in a 5% CO_2_ atmosphere. Each well was carefully washed twice after removing the supernatant by pipetting 1 ml of fresh and pre-warmed RPMI into the well along the wall of the well. Each wash was incubated for 10 min at 37°C. Subsequently the NETs were digested for 20 min in 1 ml RPMI with 10 U/ml DNase-1 (Worthington). DNase-1 was stopped with 5 mM EDTA (final concentration). The samples were centrifuged at 300×g to remove whole cells and then at 16,000×g to remove debris. Proteins were acetone precipitated. Four matched samples from different wells were pooled together and transferred to a 30 ml glass corex tube and 16 ml of ice-cold acetone (−20°C) was added. For precipitation samples were incubated overnight at −20°C and then centrifuged at 10 000×g for 30 min at 4°C. The protein pellet was washed with 1 ml 80% acetone buffered in 20 mM Tris-HCl pH 8.0 and solubilized in 120 µl SDS loading buffer or prepared for MS identification as described below.

For verification of our purification procedure we followed each step of the isolation procedure by analysis of the respective sample using silver stained SDS electrophoresis and immunoblotting. We harvested all steps including the supernatant after 4 h incubation, the washes 1 and 2, and the NET digests. As controls (i) NETs were mock-digested with nuclease-free RPMI and (ii) unstimulated neutrophils that did not release NETs were washed twice and incubated with RPMI containing 10 U/ml Dnase-1 for 20 min at 37°C. Each sample was derived from one well containing 1.7×10^6^ neutrophils in a volume of 1 ml. Four samples out of 4 wells were pooled, acetone precipitated, solubilized in 120 µl SDS loading buffer and boiled for 3 min. To account for potential protein loss due to proteolytic activity in the samples a complete purification procedure was performed in the presence of protease inhibitor cocktail (Sigma P1860; 1∶200) added to the wells 2 h after stimulation start as described above. Protease inhibitor cocktail was additionally present in all media used for washing and digestion. For analysis of the purification steps we loaded 30 µl of each pooled sample (equaling 1 well and 1.7×10^6^ neutrophils) on SDS protein gels (Tris-HCl 10–20%). The gels were either silver stained as described elsewhere [63] or immunoblotted by transferring to a PVDF membrane (Immobilon 40; Millipore). The immunoblots were performed as described in detail for the quantitative blots below. The following primary antibodies were used: α-elastase (Calbiochem 481001, 2 µg/ml), α-azurocidin (Sigma N5662, 0.5 µg/ml), α-histone H2B (Upstate 07-371, 1 µg/ml), α-S100A8 combined with α-S100A9 (Acris BM4029 and BM4027, both at 2.5 µg/ml), α-catalase (Sigma C0979, 1 µg/ml), α-GAPDH (Labfrontier LF-PA0018, 0.5 µg/ml), α-LDH (Abcam ab7639, 1 µg/ml). As secondary antibodies horseradish-peroxidase-conjugated F(ab')2 fragments (Jackson ImmunoResearch) were used in 1∶20 000 dilutions.

For MS analysis NET proteins from 3 different donors were purified independently in the absence (donor 1–3, named sample 1–3) or presence of the protease inhibitor cocktail indicated above (donor 1–2, named sample 4 and 5).

DNA-concentration, neutrophil elastase activity and reactive oxygen species (ROS) were measured as described previously [6].

### Sample preparation and digestion for MS

Samples 1–5 (acetone precipitates from NET-purifications) were solubilized in 40 µl of 500 mM triethylammonium bicarbonate buffer pH 8.5 (TEAB) and reduced with 2 µl of 50 mM tris-(2-carboxyethyl)phosphine (TCEP) for 60 min at 60°C. After alkylation with 1 µl of 200 mM methyl methanethiosulfonate (MMTS) at RT for 10 min each sample was incubated overnight at 37°C with 10 µl of a 200 µg/ml trypsin solution, solubilized in 500 mM TEAB. The reaction was stopped with 1 µl of a 10% TFA solution to obtain a final concentration of TFA of approximately 0.2%. The sample was centrifuged for 10 min at 13800×g and the supernatant used for LC/MS analysis.

### Nano-LC/MALDI-MS

The samples were analyzed by bottom-up nano-LC/MALDI-MS as described in detail in the NET Database. Proteins were digested with trypsin and the resulting peptides separated by nano-LC (Dionex). Peptides were fractionated (Probot microfraction collector, Dionex) and analyzed with a 4700 Proteomics Analyzer (Applied Biosystems) MALDI-TOF/TOF instrument. The criterion for the identification of a protein was a minimum number of 3 peptides fulfilling the Mascot homology criteria. Candidates with two peptides fulfilling these criteria were verified by checking the fragmentation rules, such as hypercleavage sites (Asp, Glu, Pro), the appearance of common immonium masses and mass losses [64]. A protein was considered as localizing to NETs only when found in at least 2 independent samples from different donors. Exceptions are MNDA, actinin and lysozyme C. MNDA and actinin were identified with one peptide in independent samples only, however the peptide is unique to both proteins within the IPI-database. Presence of lysozyme C in NETs was verified by immunoblotting. The MS analysis is described in more detail on the NET database and in the supporting materials.

### Quantification of NET proteins

NET-associated proteins were quantified by immunoblot as described [65]. Neutrophils were purified from 10 different healthy donors, proteins were quantified using the DC assay (Biorad) and DNA was quantified with Pico Green™ (Invitrogen) [6]. We isolated NET proteins as described in ‘Purification of NET-proteins’. From each donor we prepared 12 times 1.7×10^6^ neutrophils each seeded in 1 ml RPMI per well in a 12 well tissue culture plate. NETs from 10 wells were digested with 5 U/ml MNase (Fermentas), a non-processive nuclease that cuts DNA at linker sites. 2 wells were mock-digested with nuclease-free medium as controls. We measured 3 independent pools made from 3 different donors each to average samples. For each quantification, the 3 averaged samples and 6 different concentrations of the respective purified protein, used as a standard, were loaded on the same SDS-protein gel (Tris-HCl 10–20%). The gel was transferred to a PVDF-membrane (Immobilon 40; Millipore) in a liquid transfer system for 2 h at 80 V. The membrane was blocked for 1 h in PBS with 5% skim milk powder, washed 3 times in PBS with 0.05% Tween-20 (PBST). Primary antibodies (listed in the NET Database) were diluted in PBST with 1% BSA (PBST-BSA) and incubated with the membrane for 1h at room temperature or at 4°C over night. After 3 washes in PBST horseradish-peroxidase-conjugated F(ab')2 fragments (Jackson) were used as secondary antibodies and incubated for 30 min. The blots were developed with chemiluminescence and detected in a LAS 3000 camera (Fujifilm Europe). The signal intensities of the bands were analyzed by 2D densitometry (array imager software 4.15, Raytest) and the concentration calculated based on the purified protein standards in a linear range of analysis. The amount of sample was adjusted to be within this range. To confirm the reproducibility of our quantification approach, we prepared a separate batch of pooled NET proteins from additional 5 donors and quantified again 7 randomly chosen NET proteins producing comparable results (NET Database).

### Purified proteins as standards for quantitative immunoblots

Recombinant human S100A8 and S100A9 for quantification were purified as previously described [66]. cDNA of human *S100A8* and *S100A9* in the plasmid pQE32 (C. Kerkhoff) were amplified by standard PCR using primers with HindIII and NdeI restriction sites at either end (*S100A8*: AGTCCTAAGCTTCTACTCTTTGTGGCTTTCTT, ATTACACATATGATGTTGACCGAGCTGGA; *S100A9*: ATCTAACATATGATGACTTGCAAAATGTCGCAGC, ATCTTCAAGCTTTTAGGGGGTGCCCTCCC). The PCR products were cloned into the expression vector pET28a+ (Invitrogen) and confirmed by sequencing. We purchased recombinant core histones from Upstate, catalase purified from human erythrocytes (Sigma-Aldrich) and all other proteins purified from human neutrophils (Athens Research and Technology).

### High resolution FESEM analysis of NET fine structure

Human neutrophils were seeded on 12-mm cover slips and stimulated with PMA for 4 hours. After fixation (2.5% glutardialdehyde), specimens were contrasted using repeated changes of 0.5% OsO_4_ and 0.05% tannic acid. Specimens were then dehydrated in a graded ethanol series and subjected to critical point drying. After coating with platinum/carbon, specimens were analyzed in a Leo 1550 field emission scanning electron microscope (FESEM, Zeiss SMT). Peripheral NET areas with individual NET fibers were recorded at high magnification and the obtained images were analyzed using the SmartSEM software (Zeiss SMT).

### Release of calprotectin

1.7×10^6^ ml^−1^ human neutrophils were seeded in 1 ml RPMI medium containing 10 U/ml DNase-1 in 12 well tissue culture plates. For each condition to be tested three wells were seeded with neutrophils (n = 3). They were stimulated to form NETs using 20 nM PMA, or to degranulate using 5 µM f-MLP at 37°C in a 5% CO_2_ atmosphere similar to [40]. Dnase-1 was present to collect all released proteins, also the ones that are bound to NETs. At the indicated time points the supernatants were collected. Hundred µl aliquots were used to quantify LDH activity in the samples using the Cytotoxicity Assay™ (Promega) as a marker for cell death [67],[68]. As 100% control for LDH we lysed the same amount of neutrophils in 1 ml RPMI with 0.1% Triton X-100 and measured 100 µl. The rest of the samples were acetone precipitated. Precipitates were boiled in 30 µl SDS sample buffer and analyzed by immunoblotting using anti-S100A8 (Acris BM4029, 2.5 µg/ml), anti-S100A9 (Acris BM4027, 2.5 µg/ml), anti-LTF (Sigma-Aldrich L3262, 2 µg/ml) and anti-MPO (DAKO A0398, 2 µg/ml) antibodies.

To determine the relative amounts of calprotectin in the supernatant after NET formation and in NETs we seeded 1.7×10^6^ neutrophils per well and induced them to make NETs for 4 hours. We collected the supernatant and washed the cells twice. The washes were discarded. Then we digested the NETs with nuclease in the same volume and removed the supernatant again after 20 min. Subsequently, we added 200 µl reducing SDS protein sample buffer to the remaining debris in the wells and scratched them thoroughly and boiled for 3 minutes. As 100% control we lysed the same amount of neutrophils in 400 µl protein sample buffer and boiled as well. The supernatant and the NET digest were lyophilized overnight and also resuspended in 200 µl sample buffer. 40 µl of each fraction was loaded and subjected to SDS PAGE and immunoblotting with an anti-S100A9 antibody. The signal intensities of the bands were analyzed by 2D densitometry as described under “Quantification of NET proteins”. The relative amounts were calculated.

### Antimicrobial activity of NETs

5×10^5^ ml^−1^ human or murine neutrophils were stimulated with 20 nM PMA for 4 h at 37°C in a 5% CO_2_ atmosphere to form NETs in 24 well tissue culture plates. Supernatants were then removed, NETs were washed twice with 1 ml RPMI and microbes were added at a multiplicity of infection (MOI) of 0.01 if not stated differently in 500 µl of RPMI per well. Samples *C. albicans* were incubated overnight at 30°C to induce yeast-form growth or at 37°C to induce hyphal growth. NETs showed to be similarly antifungal at both temperatures (Figure S4) and purified calprotectin as well (data not shown). Assays with *C. neoformans* were incubated only at 37°C. After incubation overnight the microbes were plated on YPD agar plates to determine colony forming units (CFU) or an XTT assay was performed as shown in Figure S4. For assays in Figure 4 A & B *C. albicans* and NETs were incubated at 30°C.

### Immunodepletion of calprotectin from NETs

Twenty times 5×10^5^ human neutrophils were seeded into 24 well tissue culture plates each in 500 µl RPMI and stimulated with 20 nM PMA for 4h at 37°C in a 5% CO_2_ atmosphere to form NETs. Supernatants were removed, NETs were washed twice with 1 ml RPMI and digested with 500 µl 10 U/ml DNase-1 each. The digested NET proteins were pooled into two 5 ml fractions and concentrated 10-fold on filter columns with a 3.5 kDa cut-off to a volume of 500 µl. The concentrated samples were incubated for 2 h either with a combination of anti-S100A8 and anti-S100A9 (Acris BM4029 and BM4027, 10 µg each), or isotype mouse IgG1 as a control (Sigma M5284), immobilized on 100 µl magnetic sepharose beads (Pierce). After incubation, the samples were diluted back to the original volume using fresh medium to complement metal ions. CFU were determined after incubation of these samples overnight with *C. albicans*. Viability of *C. albicans* was also monitored using the XTT assay as stated in supporting material Figure S4. The immunodepletion was evaluated by immunoblotting. Samples were tested for the presence of calprotectin (Acris BM4027, 2.5 µg/ml) and lactotransferrin (Sigma-Aldrich L3262, 2 µg/ml) as a control.

### Co-precipitation assay of free and NET-bound calprotectin

1.7×10^6^ ml^−1^ human neutrophils were stimulated with 20 nM PMA for 4h at 37°C in a 5% CO_2_ atmosphere to form NETs in 1 ml RPMI. NET-free supernatants after incubation were removed and kept. NETs were washed twice with 1 ml RPMI and digested in 1 ml RPMI with 5 U/ml MNase for 20 min to obtain fragments of NETs. NET-free supernatants and digested NET fragments were incubated on a rolling wheel with 3×10^7^ *C. albicans* yeasts and *C. neoformans* from YPD overnight cultures previously washed 3 times in PBS. After 30 min the microbes were pelleted, the supernatants removed and washed three times in fresh medium. These new supernatants were acetone-precipitated. Precipitates from supernatants and microbial pellets were boiled in SDS sample buffer and analyzed by immunoblotting using anti-S100A8 and anti-S100A9 antibodies (Acris BM4029 and BM4027, both at 2.5 µg/ml) as described above.

### Infections and immunohistochemistry

For analysis of abscess formation 10 anesthetized animals per group were shaved on the back and subcutaneously infected with 5×10^7^ *C. albicans*. Abscesses were measured with a caliper at the indicated time points. Pulmonary candidiasis was induced by intranasal challenge of anesthetized animals with 5×10^7^ *C. albicans* and systemic candidiasis by intravenous injection to the tail vein with 5×10^5^ *C. albicans* in 10 animals per group. Inoculation doses were determined by CFU counts after plating. For intranasal and intravenous challenge, survival was monitored daily. For fungal load, lungs from 11 mice per group were macerated and CFU determined 3 days after intranasal challenge. For histology of pulmonary infections lungs were removed 24 h after intranasal challenge and 6 days after subcutaneous challenge abscesses were removed. Tissue samples were fixed in 2% formalin, dehydrated, embedded in paraffin, sliced to 5 µm, rehydrated and stained with hematoxylin and eosin (H & E). For immunostainings, samples were rehydrated, subjected to antigen retrieval and incubated with primary antibodies directed against calprotectin subunits S100A8 and S100A9 (produced in house), MPO (DAKO A0398) and histone (Santa Cruz 8030). These were detected with secondary antibodies coupled to Cy2, Cy3 or Cy5. DNA was detected with DRAQ5™ (Biostatus).

For fine structural analysis of paraffin-embedded tissue samples, 5 µm sections were rehydrated, postfixed with glutaraldehyde, contrasted using repeated changes of 0.5% OsO_4_ and 0.05% tannic acid, dehydrated in a graded ethanol series, critical-point dried and coated with 5 nm platinum/carbon.

For immunostainings with human neutrophils, 1×10^5^ cells were seeded on 13 mm glass cover slips, stimulated with 20 nM PMA and fixed in 2% formalin at the indicated time points. Specimens were blocked with 3% cold water fish gelatin, 5% donkey serum, 1% BSA, 0.25% TWEEN 20 in PBS, incubated with primary antibodies directed against S100A8/A9 complex (Acris BM4025) and MPO (DAKO A0398) and then washed. Primary antibodies were detected with species-specific secondary antibodies and DNA with DRAQ5™. Specimens were analyzed using a SP5 confocal microscope (Leica).

### Statistical analysis

One-way analysis of variance (ANOVA) with Bonferroni post-tests was applied when multiple groups were compared and two-tailed Student's t-test was used for analysis of two groups. For non-parametrically distributed data, the two-tailed Mann-Whitney test was used. Survivals of infected mice were determined by the log-rank test. Differences were considered statistically significant at P<0.05. All statistical tests were performed using GraphPad Prism version 4.02.

## Supporting Information

Figure S1 Optimization of NET protein purification. Human neutrophils were induced to make NETs. (A) The optimal time point for isolation of NET proteins was determined by monitoring DNA amount and neutrophil elastase (NE) activity at the indicated time points. Four hours after stimulation, both DNA amount and NE activity reached a maximum. This time point was chosen for purification and identification of NET proteins. (B–C) Different nucleases were compared. DNase-1 (B) and MNase (C), a non-processive nuclease, digest NETs to release a maximum of 2.5 µg/ml DNA. Thus we used 10 U/ml Dnase-1 for protein identifications. For a stable DNA concentration in all quantitative analyses we used 5 U/ml MNase and normalized protein concentration to DNA concentration. NETs were digested for different amounts of time. Both Dnase-1 (D) and MNase (E) released a maximum of DNA before 10 min of digest. To ensure complete degradation of NETs 20 min were used for all experiments. The concentration (F) of Dnase-1, as well as the time of digest (G), was confirmed in a silver-stained SDS-PAGE analysis to be optimal at 10 U/ml and 20 min for a maximal protein yield. Shown are means±s.d. (n = 3) of representative experiments from two.
(4.40 MB TIF)

Figure S2 Mass spectrometry (MS) identification quality and immunoassays to verify the absence or presence of proteins in NETs. (A) Representative identification of NET protein S100A9 by LC-MS/MS. We obtained 51% sequence coverage from 8 MS/MS spectra and a Mascot ion score of 102. We show a representative MS/MS spectrum of one peptide mass (1806.9532). In this case, identification was confirmed by 9 y-ions in series and 11 b-ions, and the immonium ion of His (H). We evaluated the NET association of (B) bactericidal/permeability increasing protein (BPI), (C) pentraxin 3 (PTX-3) and (D) cathelicidin CAP-18 that have been described as NET-associated [3],[4],[37],[38], but were not found in our MS approach. BPI and CAP-18 are endogenously cleaved by neutrophil proteinases into a 25 kDa and a 5 kDa (LL-37) cleavage product [2]. We immunoblotted neutrophil (PMN) lysates, NET extracts and purified proteins as positive controls. Human neutrophil granular extract (hNGE) was used as positive control for the presence of BPI, as well as recombinant human PTX-3 and purified human LL-37 peptide. We confirmed NET association of BPI, but not of PTX-3 and CAP-18. (E) Quantification of calprotectin. We confirmed that calprotectin was a bona fide NET protein by washing the NETs until unbound calprotectin was not detectable using an enzyme-linked immunosorbent assay (ELISA) from Hycult. The data are the average of two independent experiments (means±s.d., n = 4), bdl = below detection limit (1.6 ng/ml).
(0.42 MB TIF)

Figure S3 NET Protein quantification. Representative quantitative immunoblots for S100A8 (A) and MPO (E) showing six different protein amounts of the standard protein as well as a NET sample in triplicate. The signal intensities (B and F) were plotted against the amount of standard protein within a linear range (C for S100A8 and G for MPO). The resulting equation was used to calculate the respective amounts of proteins as means from three different samples (D and H). The protein amounts (D and H) are specified as mg or µmoles referred to the amount of DNA within the sample. The DNA amount is the mean of all samples used for this quantification. One sample equals the amount of NETs isolated from 1.7×10^6^ human neutrophils. Similar analyses of all the quantified proteins are available on the NET database.
(0.98 MB TIF)

Figure S4 Antifungal activity and composition of NETs is similar under different conditions (MOI or temperature) and stimuli (PMA or *C. albicans*). Human neutrophils were induced to make NETs and washed twice. *C. albicans* and NETs were incubated overnight in all assays either at 30°C to preserve yeast-form growth or at 37°C to induce hyphal growth. (A) *Cryptococcus neoformans* or *C. albicans* were added to NETs with a MOI of 0.04, incubated overnight at 37°C and CFU determined, for C.n.+/−NETs P<0.01, for C.a.+/−NETs P<0.05. (B) C. albicans was added to NETs and incubated to induce hyphal growth. NETs inhibit *C. albicans* hyphae at a MOI of 0.04 and 0.01 similarly. (C) *C. albicans* was added to NETs and incubated to preserve yeast-form growth. NETs inhibit *C. albicans* yeast at a MOI of 0.2, 0.04 and 0.01 similarly. (D) Addition of MNase to NETs significantly reduced inhibition of *C. albicans* yeast, P<0.01. (A–D) Shown are means±s.d. (n = 3) from one representative experiment out of three. (E) Representative tissue culture plate and (F) light microscopic image with growing *C. albicans* hyphae in the absence (top) or presence of NETs (bottom). (G–I) Antifungal NET assays using XTT [46] we confirmed that NETs also reduce *C. albicans* growth under hyphae inducing conditions at 37°C only in the absence of 0.5 µM Zn^2+^ for +/−NETs at 0 µM Zn^2+^ P<0.001 (G). NET protein extracts inhibited hyphal growth significantly stronger than calprotectin-depleted extracts, for +/− depletion P<0.01 (H). NETs from wild-type mouse neutrophils reduced hyphal growth 20-fold whereas S100A9-knockout neutrophils only reduced growth 3-fold, for WT vs. KO P<0.001 (I). Shown are means±s.d. (n = 3) from one experiment out of two. (J) Human neutrophils were induced to make NETs overnight using either 20 nM PMA or *C. albicans* (MOI 2) in the presence of protease inhibitors. NET proteins were purified and protein concentrations of samples were determined and equal amounts loaded. Immunoblots probed for lactotransferrin (LTF), cathepsin G (CG), calprotectin and catalase indicated a similar protein composition of NETs independent of the stimulus. Differences in amounts of individual proteins might be caused by an overall higher protein concentration in samples from *C. albicans* induced NETs due to secreted *C. albicans* proteins.
(4.12 MB TIF)

Figure S5 Calprotectin-deficient mice release similar amounts of NETs as wild-type mice *in vitro* and *in vivo*. (A) NET-DNA release was induced with PMA and measured by Sytox Green™. There were no significant differences between calprotectin-deficient and wild type mouse neutrophils. (B) H & E stain of an abscess section from calprotectin-deficient mice, 6 days after infection. NETs are indicated by arrows, scale bar = 50µm. (C–F) Confocal images of indirect immunofluorescence with antibodies against (C) S100A9 (red), (D) MPO (green), (E) histone (blue) and (F) superimposition of all signals. Arrows indicate NETs. No stain can be detected for the anti-S100A9 antibody verifying its specificity. Scale bar = 50 µm. (G) Neutrophil recruitment was counted from images of representative abscess sections for calprotectin-deficient and wild type mice and calculated as neutrophils/area (mm^2^). Shown are means μ s.d. of triplicates from three independent abscesses per group.
(4.10 MB TIF)

Figure S6 *C. albicans* infection spread after subcutaneous challenge in calprotectin-deficient but not in wild type mice. In calprotectin-deficient mice, abscess lesions spread to other locations in approximately 30% of the infected animals (n = 10). Spreading did not occur in wild type animals. (A) Shown are representative abscesses from a calprotectin-deficient and (B) from a wild type mouse at day 21 p.i.
(5.00 MB TIF)

Text S1 Supporting Methods
(0.05 MB DOC)
