# Supplementary figures and images for: Neutrophil Extracellular Traps Contain Calprotectin, a Cytosolic Protein Complex Involved in Host Defense against Candida albicans
Source: PLoS Pathog. 2009 Oct 30;5(10):e1000639. doi: 10.1371/journal.ppat.1000639 (PMC2763347; doi:10.1371/journal.ppat.1000639)

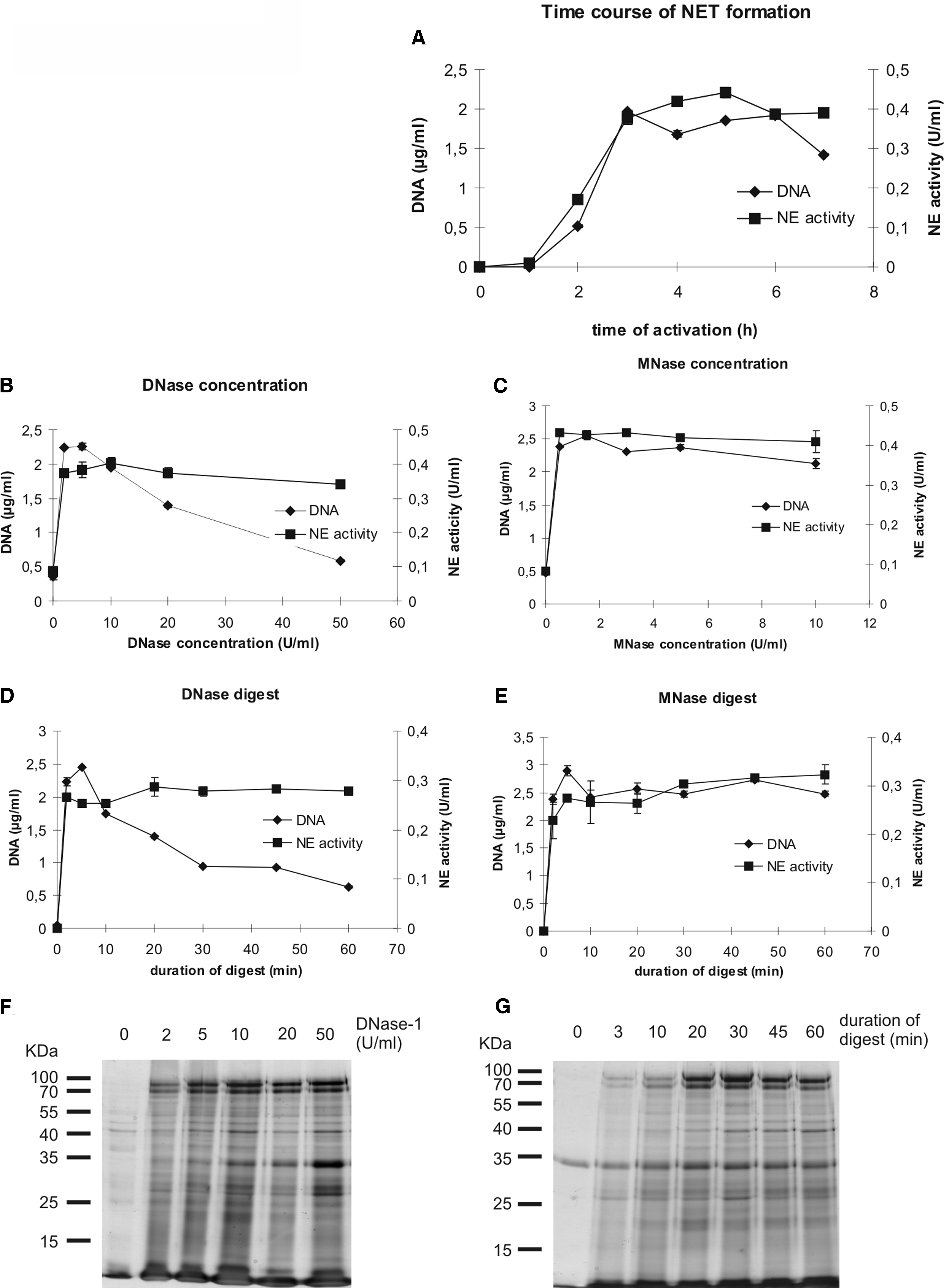

Supplement: Figure S1 — Optimization of NET protein purification. Human neutrophils were induced to make NETs. (A) The optimal time point for isolation of NET proteins was determined by monitoring DNA amount and neutrophil elastase (NE) activity at the indicated time points. Four hours after stimulation, both DNA amount and NE activity reached a maximum. This time point was chosen for purification and identification of NET proteins. (B–C) Different nucleases were compared. DNase-1 (B) and MNase (C), a non-processive nuclease, digest NETs to release a maximum of 2.5 µg/ml DNA. Thus we used 10 U/ml Dnase-1 for protein identifications. For a stable DNA concentration in all quantitative analyses we used 5 U/ml MNase and normalized protein concentration to DNA concentration. NETs were digested for different amounts of time. Both Dnase-1 (D) and MNase (E) released a maximum of DNA before 10 min of digest. To ensure complete degradation of NETs 20 min were used for all experiments. The concentration (F) of Dnase-1, as well as the time of digest (G), was confirmed in a silver-stained SDS-PAGE analysis to be optimal at 10 U/ml and 20 min for a maximal protein yield. Shown are means±s.d. (n = 3) of representative experiments from two. (4.40 MB TIF) [file ppat.1000639.s001.tif]

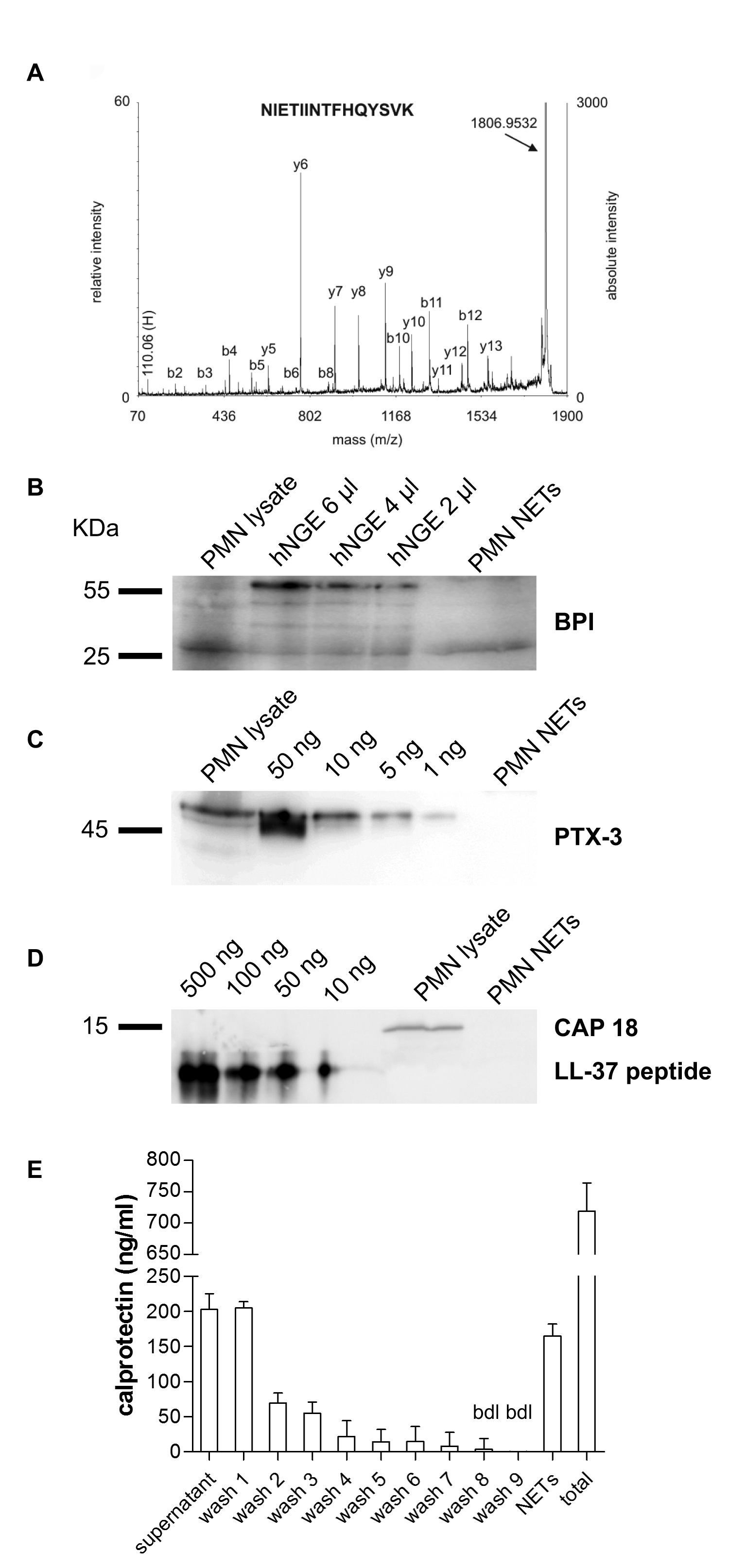

Supplement: Figure S2 — Mass spectrometry (MS) identification quality and immunoassays to verify the absence or presence of proteins in NETs. (A) Representative identification of NET protein S100A9 by LC-MS/MS. We obtained 51% sequence coverage from 8 MS/MS spectra and a Mascot ion score of 102. We show a representative MS/MS spectrum of one peptide mass (1806.9532). In this case, identification was confirmed by 9 y-ions in series and 11 b-ions, and the immonium ion of His (H). We evaluated the NET association of (B) bactericidal/permeability increasing protein (BPI), (C) pentraxin 3 (PTX-3) and (D) cathelicidin CAP-18 that have been described as NET-associated [3],[4],[37],[38], but were not found in our MS approach. BPI and CAP-18 are endogenously cleaved by neutrophil proteinases into a 25 kDa and a 5 kDa (LL-37) cleavage product [2]. We immunoblotted neutrophil (PMN) lysates, NET extracts and purified proteins as positive controls. Human neutrophil granular extract (hNGE) was used as positive control for the presence of BPI, as well as recombinant human PTX-3 and purified human LL-37 peptide. We confirmed NET association of BPI, but not of PTX-3 and CAP-18. (E) Quantification of calprotectin. We confirmed that calprotectin was a bona fide NET protein by washing the NETs until unbound calprotectin was not detectable using an enzyme-linked immunosorbent assay (ELISA) from Hycult. The data are the average of two independent experiments (means±s.d., n = 4), bdl = below detection limit (1.6 ng/ml). (0.42 MB TIF) [file ppat.1000639.s002.tif]

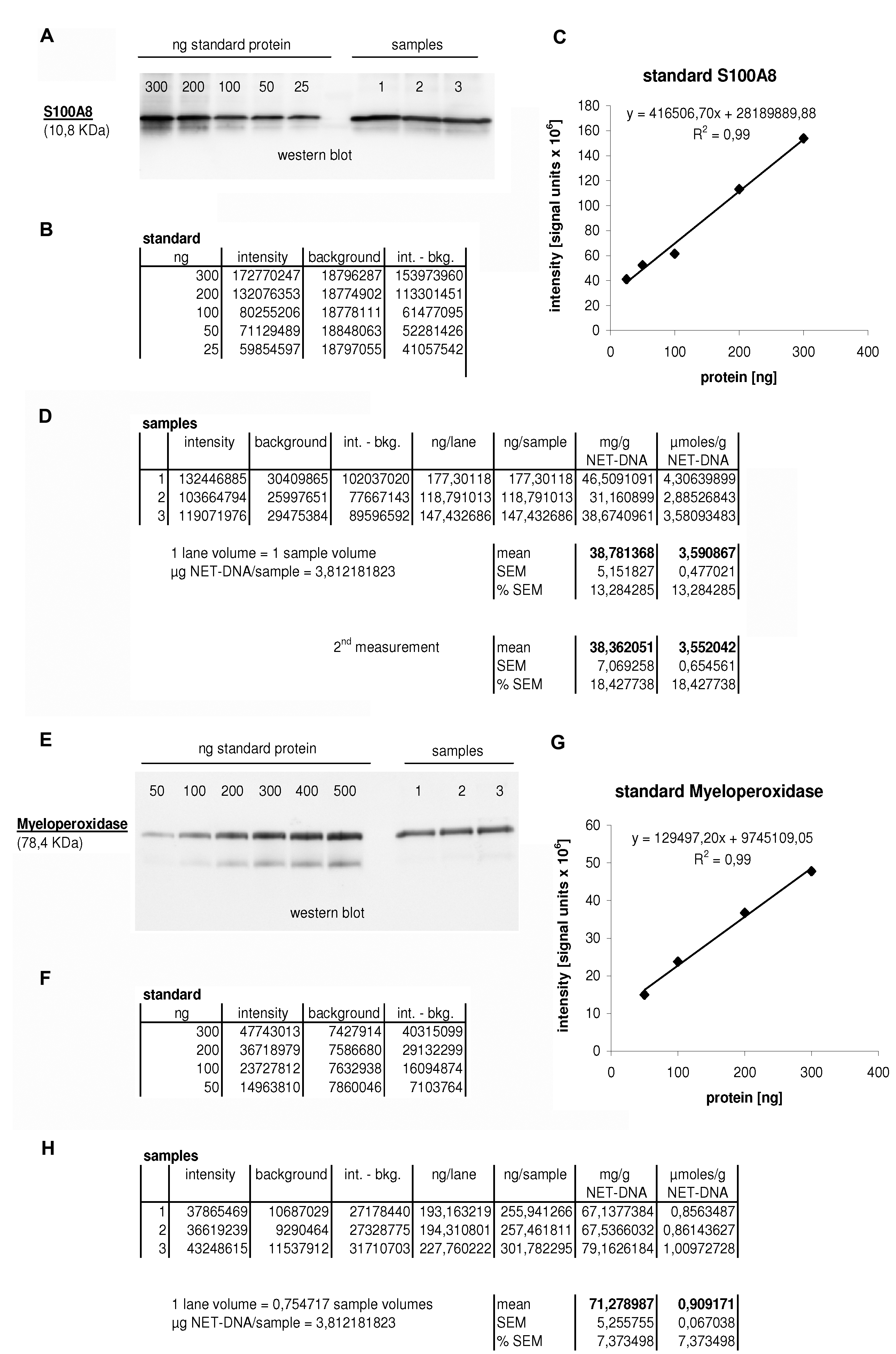

Supplement: Figure S3 — NET Protein quantification. Representative quantitative immunoblots for S100A8 (A) and MPO (E) showing six different protein amounts of the standard protein as well as a NET sample in triplicate. The signal intensities (B and F) were plotted against the amount of standard protein within a linear range (C for S100A8 and G for MPO). The resulting equation was used to calculate the respective amounts of proteins as means from three different samples (D and H). The protein amounts (D and H) are specified as mg or µmoles referred to the amount of DNA within the sample. The DNA amount is the mean of all samples used for this quantification. One sample equals the amount of NETs isolated from 1.7×10^6^ human neutrophils. Similar analyses of all the quantified proteins are available on the NET database. (0.98 MB TIF) [file ppat.1000639.s003.tif]

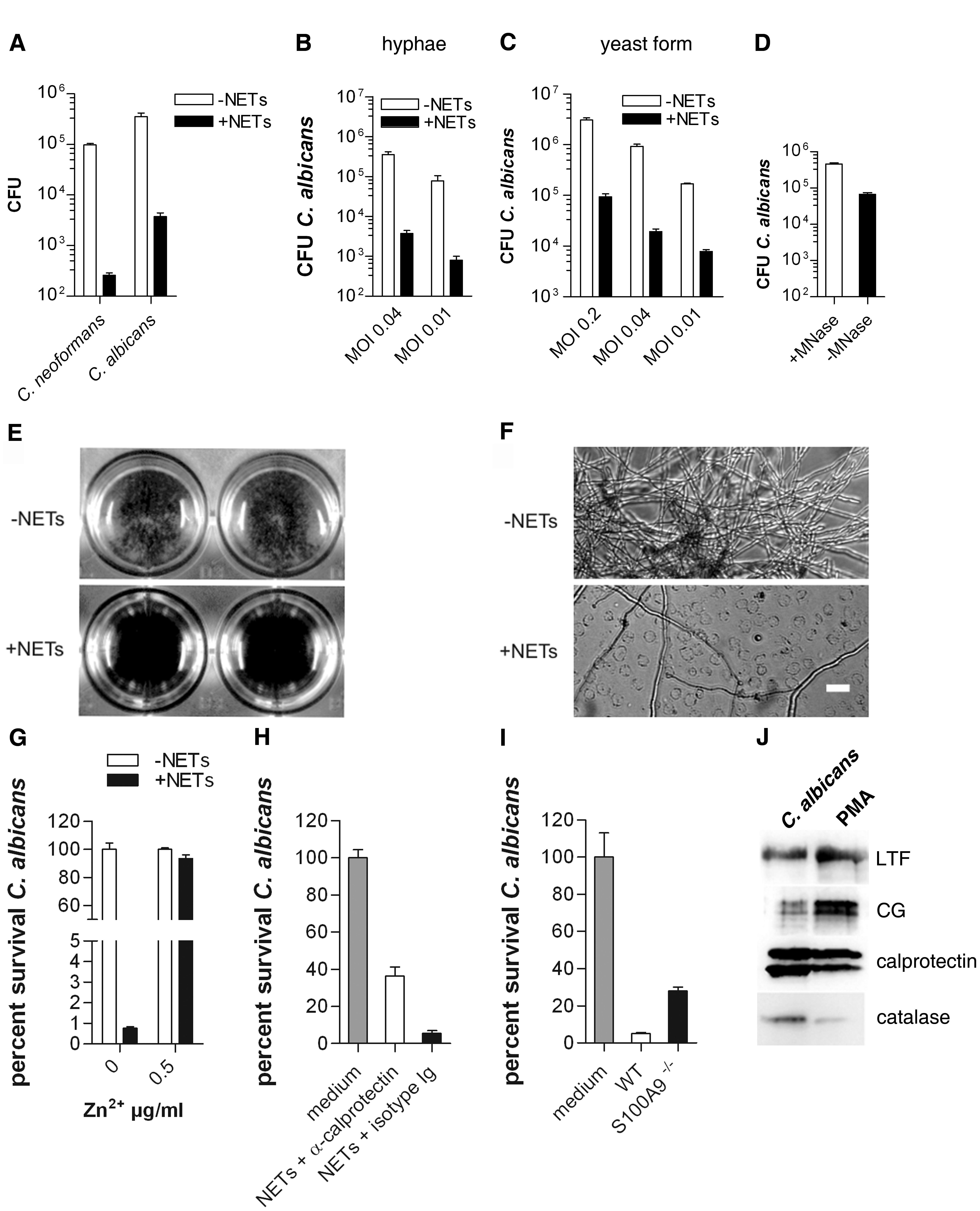

Supplement: Figure S4 — Antifungal activity and composition of NETs is similar under different conditions (MOI or temperature) and stimuli (PMA or C. albicans). Human neutrophils were induced to make NETs and washed twice. C. albicans and NETs were incubated overnight in all assays either at 30°C to preserve yeast-form growth or at 37°C to induce hyphal growth. (A) Cryptococcus neoformans or C. albicans were added to NETs with a MOI of 0.04, incubated overnight at 37°C and CFU determined, for C.n.+/−NETs P<0.01, for C.a.+/−NETs P<0.05. (B) C. albicans was added to NETs and incubated to induce hyphal growth. NETs inhibit C. albicans hyphae at a MOI of 0.04 and 0.01 similarly. (C) C. albicans was added to NETs and incubated to preserve yeast-form growth. NETs inhibit C. albicans yeast at a MOI of 0.2, 0.04 and 0.01 similarly. (D) Addition of MNase to NETs significantly reduced inhibition of C. albicans yeast, P<0.01. (A–D) Shown are means±s.d. (n = 3) from one representative experiment out of three. (E) Representative tissue culture plate and (F) light microscopic image with growing C. albicans hyphae in the absence (top) or presence of NETs (bottom). (G–I) Antifungal NET assays using XTT [46] we confirmed that NETs also reduce C. albicans growth under hyphae inducing conditions at 37°C only in the absence of 0.5 µM Zn2+ for +/−NETs at 0 µM Zn2+ P<0.001 (G). NET protein extracts inhibited hyphal growth significantly stronger than calprotectin-depleted extracts, for +/− depletion P<0.01 (H). NETs from wild-type mouse neutrophils reduced hyphal growth 20-fold whereas S100A9-knockout neutrophils only reduced growth 3-fold, for WT vs. KO P<0.001 (I). Shown are means±s.d. (n = 3) from one experiment out of two. (J) Human neutrophils were induced to make NETs overnight using either 20 nM PMA or C. albicans (MOI 2) in the presence of protease inhibitors. NET proteins were purified and protein concentrations of samples were determined and equal amounts loaded. Immunoblots probed for lactotransferrin [file ppat.1000639.s004.tif]

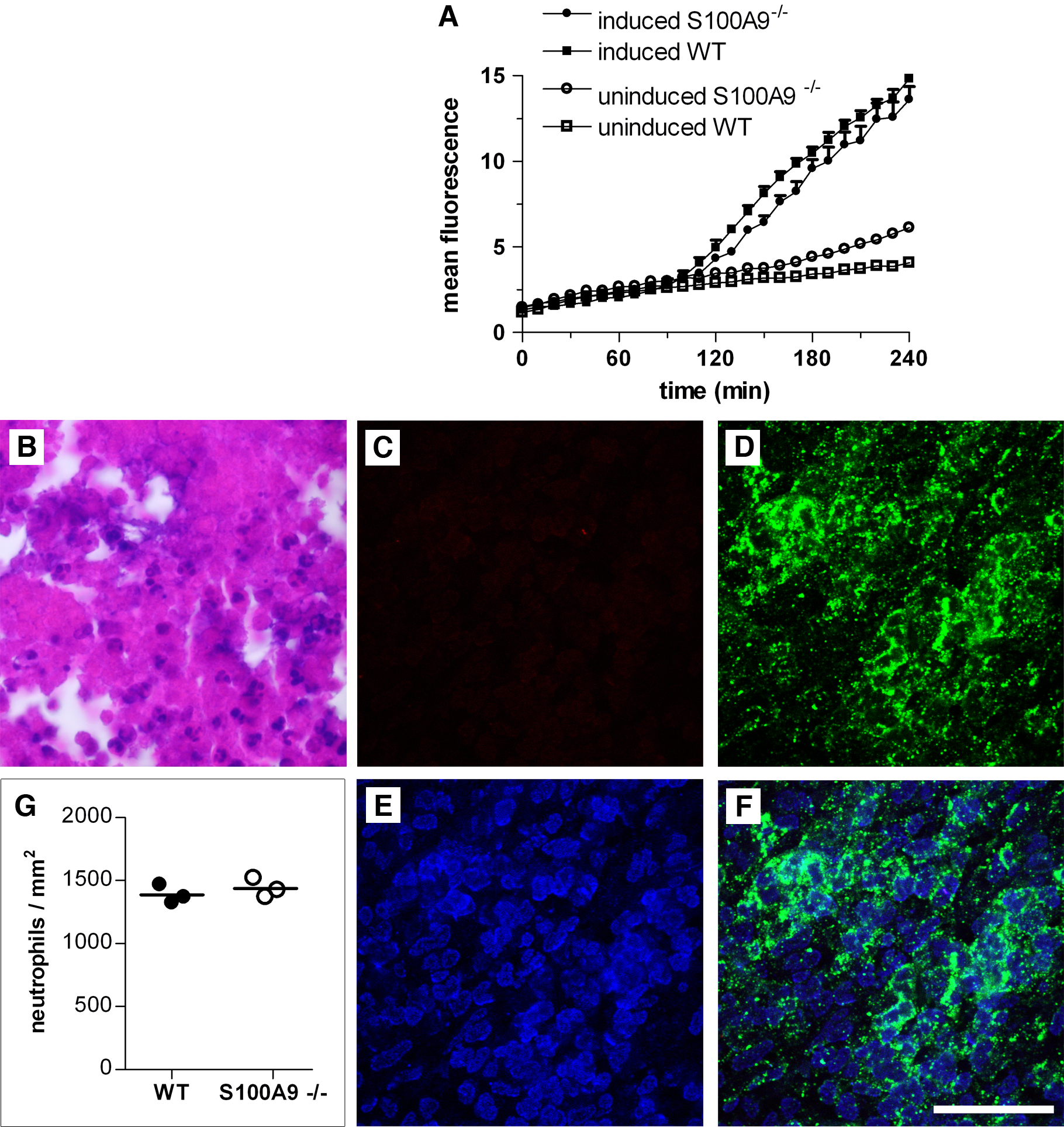

Supplement: Figure S5 — Calprotectin-deficient mice release similar amounts of NETs as wild-type mice in vitro and in vivo. (A) NET-DNA release was induced with PMA and measured by Sytox Green™. There were no significant differences between calprotectin-deficient and wild type mouse neutrophils. (B) H & E stain of an abscess section from calprotectin-deficient mice, 6 days after infection. NETs are indicated by arrows, scale bar = 50µm. (C–F) Confocal images of indirect immunofluorescence with antibodies against (C) S100A9 (red), (D) MPO (green), (E) histone (blue) and (F) superimposition of all signals. Arrows indicate NETs. No stain can be detected for the anti-S100A9 antibody verifying its specificity. Scale bar = 50 µm. (G) Neutrophil recruitment was counted from images of representative abscess sections for calprotectin-deficient and wild type mice and calculated as neutrophils/area (mm2). Shown are means μ s.d. of triplicates from three independent abscesses per group. (4.10 MB TIF) [file ppat.1000639.s005.tif]

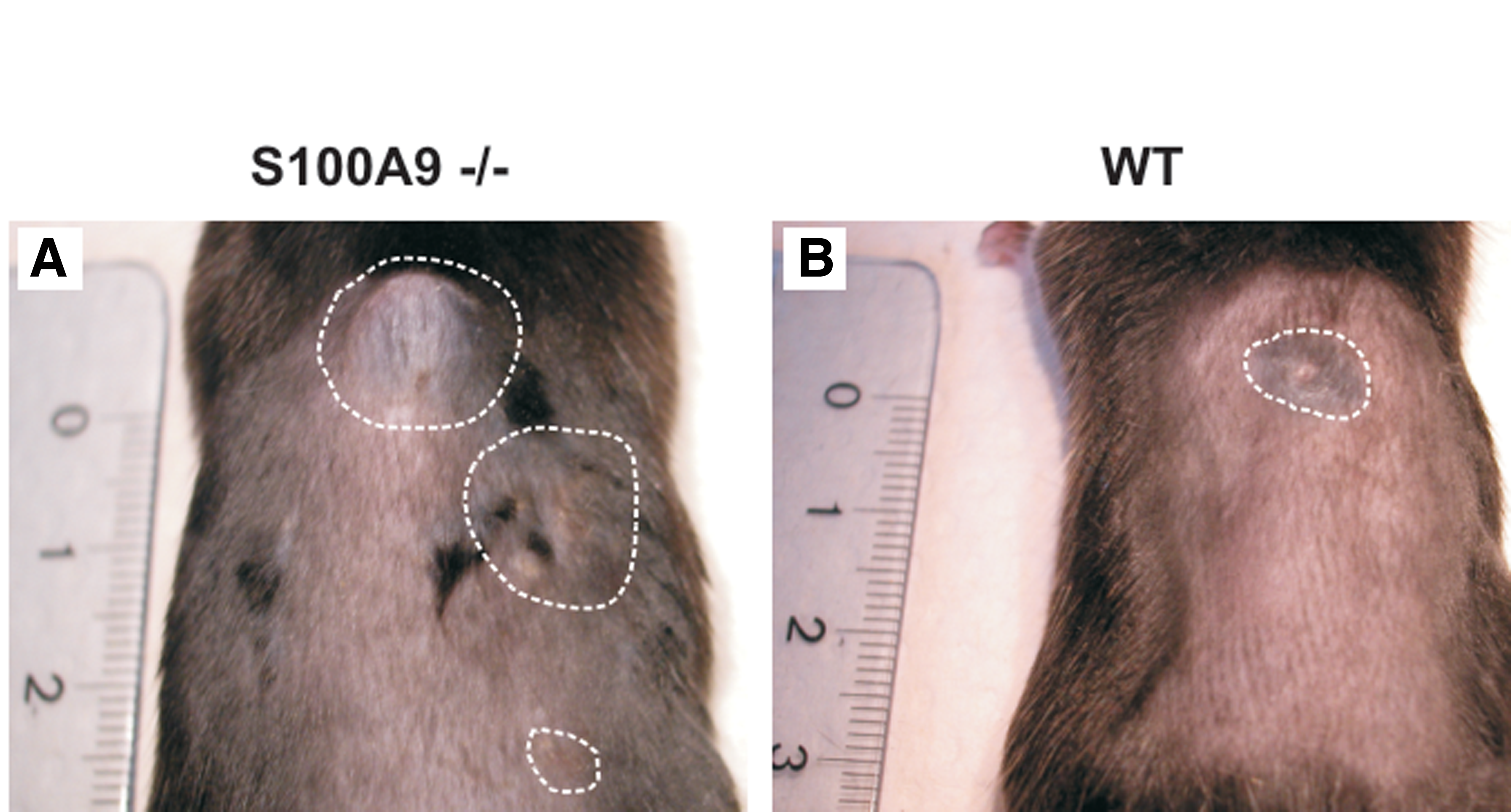

Supplement: Figure S6 — C. albicans infection spread after subcutaneous challenge in calprotectin-deficient but not in wild type mice. In calprotectin-deficient mice, abscess lesions spread to other locations in approximately 30% of the infected animals (n = 10). Spreading did not occur in wild type animals. (A) Shown are representative abscesses from a calprotectin-deficient and (B) from a wild type mouse at day 21 p.i. (5.00 MB TIF) [file ppat.1000639.s006.tif]
