# Supplementary material for: Neutrophil Extracellular Traps Contain Calprotectin, a Cytosolic Protein Complex Involved in Host Defense against Candida albicans
Source: PLoS Pathog. 2009 Oct 30;5(10):e1000639. doi: 10.1371/journal.ppat.1000639 (PMC2763347; doi:10.1371/journal.ppat.1000639)
Supplement: Text S1 — Supporting Methods (0.05 MB DOC) [file ppat.1000639.s007.doc]

**Supporting Methods (Urban *et al*.)**

**Optimization of NET protein purification**

1.7 × 10^6^ ml^-1^ human neutrophils were induced to make NETs using 20 nM PMA. Samples were collected: (i) At different time points after PMA activation and digested with Dnase-1 (Worthington) or MNase (Fermentas) with both (ii) different concentrations and (iii) different incubation times. From those samples neutrophil elastase (NE) activity and extracellular DNA concentration was measured as described [1]. Samples were also prepared for a silver-stained SDS-PAGE 10 – 20 % Tris-HCl. Silver staining was performed as described [2].

**Detailed description of Nano-LC/MALDI-MS**

Each peptide mixture was diluted 1:5 with 0.1% TFA and centrifuged for 10 min at 13800 x g. 10 µl of the supernatant were then fractionated by nano-LC on an Ultimate system (Dionex, Sunnyvale, CA). The peptides were desalted and concentrated with a PepMap C18, 5 µm, 100 Å precolumn (300 µm I.D. x 5 mm, Dionex) at a flow rate of 20 µl/min and fractionated with a PepMap C18, 3 µm, 100 Å (75 µm I.D. x 150 mm, Dionex) at a flow rate of 200 nl/min. The gradient was built up with H_2_O/ACN/TFA (98:2:0.05, v/v/v) as solvent A and H_2_O/ACN/TFA (20:80:0.045, v/v/v) as solvent B. The elution gradients were 0-15% B for 5 min, 15-60% B for 60 min, 60-100% B for 5 min, 100% B for 5 min and 100-0% B for 8 min. The UV chromatogram was recorded at a wavelength of 214 nm. Peptide fractionation was performed with a Probot micro fraction collector (Dionex). Alpha- Cyano-4-hydroxycinnamic acid (Aldrich 1302B1, recrystallized and stored at -20 °C) at a concentration of 5mg/ml and dissolved in H2O/ACN/TFA (30:70:0.1, v/v/v) was used as matrix. The matrix was added to the eluent flow via a micro-T-piece (Upchurch, Oak Harbor, WA) at a flow rate of 1 µl/min. Spotting was started 25 min after initiation of the elution gradient at 10 second intervals on a 4700 Sample Plate. A total of 288 fractions were spotted for each sample. MALDI-MS was performed using a 4700 Proteomics Analyzer (Applied Biosystems) MALDI-TOF/TOF instrument. MS spectra were recorded with 1200 shots per spot and processed with default calibration. Mass calibration of the plate was obtained by spotting a mixture of reference peptides (des-Arg-1-bradykinin, MH = 904.468 Da; angiotensin I, MH = 1296.685 Da; Glu1-fibrinopeptide, MH =1570.677 Da; ACTH (1-17), MH = 2093.087 Da; ACTH (18-39), MH = 2465.199 Da; ACTH (1-39)) at six places on the left and right side of the spotted samples. MS/MS precursor selection was performed automatically, using the 4000 Series Explorer Software 3.6 with the following parameters: a minimum S/N ratio of 40 and a maximum of 5 MS/MS per spot. MS/MS spectra were acquired without CID and with stop conditions (2500 to 10000 shots). This description is also presented on the NET database (http://141.14.152.84/cgi-bin/36525/pdbs/lc/index.cgi).

**Immunoblots for BPI, PTX-3 and LL-37**

We immunoblotted neutrophil lysates, NET extracts and as positive controls purified proteins. Neutrophil lysates were obtained by boiling 3 × 10^6^ cells in protein sample buffer. NET proteins were purified as described in ‘Materials and Methods’. Human neutrophil granular extract (hNGE), prepared as previously described [3], was used as positive control for the presence of BPI. Recombinant human PTX-3 (Alexis) and purified human LL-37 peptide, a cleavage product from CAP-18 (Hycult) were used as controls as well. SDS-PAGE and blotting was performed as described in ‘Materials and Methods’. The following primary antibodies were used: α-BPI (Hycult, HP9022, 10 µg/ml), α-pentraxin-3 (Alexis ALX-804-464, 5 µg/ml) and α-LL-37 (Hycult, HM2071, 10 µg/ml)

**Calprotectin ELISA**

Calprotectin concentrations in supernatants after NET formation, 9 consecutive washes and nuclease-digested NETs were quantified by ELISA (Hycult, HK325) following the manufacturer’s instructions. We used the same approach to isolate NET proteins as described in ‘Materials and Methods’. Briefly, we induced 1.7 × 10^6^ neutrophils in 1 ml RPMI to release NETs using 20 nM PMA for 4h at 37 °C with 5 % CO_2_. We collected the supernatant and washed each well 9 times carefully by pipetting 1 ml of fresh and pre-warmed RPMI into the well along the wall of the well. Each wash was incubated for 10 min at 37 °C. Each wash was collected. Subsequently, we digested the NETs using 1 ml RPMI with 5 U/ml MNase for 20 min. The NET digest was also collected. All samples were prepared in duplicates. As a control for the total amount of calprotectin present we used as well 1.7 x 10^6^ neutrophils per well, induced them to make NETs, collected the supernatant, added new medium with nuclease and scratched the same well with a cell scraper to remove all neutrophil remnants from the bottom of the well and pooled both supernatant and digest. We controlled cell lysis microscopically with trypan blue staining and determined that more than 95 % of the cells were disrupted by this procedure. Then we centrifuged the sample at 10 000 g for 10 minutes to remove the debris. 100 µl from each sample was diluted 1/5, 1/10 and 1/40 and measured by ELISA.

**Antifungal activity of NETs**

5 x 10^5^ ml^-1^ human or murine neutrophils were stimulated with 20 nM PMA for 4 h at 37 °C in a 5% CO_2_ atmosphere to form NETs in 24 well tissue culture plates. Supernatants were then removed, NETs were washed twice with 1 ml RPMI and microbes were added at a multiplicity of infection (MOI) of 0.01, 0.04 or 0.2 in 500 µl of RPMI per well. The samples were incubated overnight at 30 °C to induce yeast-form growth or at 37 °C to induce hyphal growth. Medium was carefully removed from all wells without removing *C. albicans*. 500 µl deionized H_2_O was added and *C. albicans* were harvested. Each well was washed again with 500 µl PBS and the bottoms of the wells were thoroughly scratched with a pipette tip until no *C. albicans* remained. The 500 µl PBS suspension was pooled with the according H_2_O sample and serially diluted in PBS. The dilutions were plated on YPD agar plates, incubated for 24h at 30 °C and colony forming units (CFU) were counted. To remove NETs prior to incubation with *C. albicans*, NETs were digested with 1 ml RPMI containing 5 U/ml MNase and remnants were washed twice with 1 ml RPMI. *C. albicans* were added at MOI of 0.01 and incubated overnight at 37 °C. For macro- and microscopic investigation of the antifungal NET assays we prepared NETs as described above and added *C. albicans* at a MOI of 0.01. The tissue culture plates were incubated overnight at 37 °C and examined with an imaging system (Alpha Imager) and by light microscopy (Leica).

Additionally viability of *C. albicans* was assessed using the the tetrazolium dye XTT [4]. The assays were performed as described above, NETs were washed twice with 1 ml RPMI and *C. albicans* were added at a MOI of 0.01 in 500 µl of RPMI per well. Samples were incubated overnight at 37 °C. Medium was carefully removed from all wells without removing *C. albicans*. H_2_O (500 µl) was added to each well, incubated for 5 min and removed again. Subsequently, 400 µl of a freshly prepared 0.5 mg/ml XTT (Invitrogen) solution in PBS with 40 µg/ml coenzyme Q_0_ (Sigma) was added to each well and incubated at 37 °C for 30 min. From each sample 100 µl were transferred to a 96 well plate and measured at 450 nm using a micro plate reader (BMG Labtech).

**Mouse NETs**

NET-DNA release was induced with PMA and measured by Sytox Green^TM^ detection for 4 h as described previously [5]. Immunohistochemistry of sections from *C. albicans* subcutaneous murine abscesses were done as described in ‘Materials and Methods’. Tissue samples (6 days p.i.) were fixed in 2% formalin, dehydrated, embedded in paraffin, sliced to 5 μm, rehydrated and stained with hematoxylin and eosin (H & E). For immunostainings, samples were rehydrated, subjected to antigen retrieval and incubated with primary antibodies directed against calprotectin subunits S100A9 (produced in house, 10 µg/ml), MPO (DAKO A0398, 10 µg/ml) and histone (Santa Cruz 8030 10 µg/ml). Neutrophil recruitment was counted using images from abscess sections and calculated as neutrophils/area (mm^2^). Three representative areas were chosen from three different abscesses from each group of mice and neutrophils were counted in H & E stains as judged by nuclear morphology.

**References for supporting methods**

1. Fuchs TA, Abed U, Goosmann C, Hurwitz R, Schulze I, et al. (2007) Novel cell death program leads to neutrophil extracellular traps. J Cell Biol 176: 231-241.

2. Zimny-Arndt U, Schmid M, Ackermann R, Jungblut PR (2009) Classical proteomics: two-dimensional electrophoresis/MALDI mass spectrometry. Methods Mol Biol 492: 65-91.

3. Weinrauch Y, Drujan D, Shapiro SD, Weiss J, Zychlinsky A (2002) Neutrophil elastase targets virulence factors of enterobacteria. Nature 417: 91-94.

4. Meshulam T, Levitz SM, Christin L, Diamond RD (1995) A simplified new assay for assessment of fungal cell damage with the tetrazolium dye, (2,3)-bis-(2-methoxy-4-nitro-5-sulphenyl)-(2H)-tetrazolium-5-carboxanil ide (XTT). J Infect Dis 172: 1153-1156.

5. Ermert D, Urban CF, Laube B, Goosmann C, Zychlinsky A, et al. (2009) Mouse Neutrophil Extracellular Traps in Microbial Infections. J Innate Immun 1: 181-193.
